# Carboxysome Mispositioning Alters Growth, Morphology, and Rubisco Level of the Cyanobacterium Synechococcus elongatus PCC 7942

**DOI:** 10.1128/mBio.02696-20

**Published:** 2021-08-03

**Authors:** Rees Rillema, Y Hoang, Joshua S. MacCready, Anthony G. Vecchiarelli

**Affiliations:** a Department of Molecular, Cellular, and Developmental Biology, University of Michigan, Ann Arbor, Michigan, USA; University of California, Berkeley

**Keywords:** Rubisco, carbon dioxide assimilation, carbon dioxide concentration mechanism, carbon dioxide fixation, carboxysome, cell division, cyanobacteria, filamentation

## Abstract

Cyanobacteria are the prokaryotic group of phytoplankton responsible for a significant fraction of global CO_2_ fixation. Like plants, cyanobacteria use the enzyme ribulose 1,5-bisphosphate carboxylase/oxidase (Rubisco) to fix CO_2_ into organic carbon molecules via the Calvin-Benson-Bassham cycle. Unlike plants, cyanobacteria evolved a carbon-concentrating organelle called the carboxysome—a proteinaceous compartment that encapsulates and concentrates Rubisco along with its CO_2_ substrate. In the rod-shaped cyanobacterium Synechococcus elongatus PCC 7942, we recently identified the McdAB system responsible for uniformly distributing carboxysomes along the cell length. It remains unknown what role carboxysome positioning plays with respect to cellular physiology. Here, we show that a failure to distribute carboxysomes leads to slower cell growth, cell elongation, asymmetric cell division, and elevated levels of cellular Rubisco. Unexpectedly, we also report that even wild-type S. elongatus undergoes cell elongation and asymmetric cell division when grown at the cool, but environmentally relevant, growth temperature of 20°C or when switched from a high- to ambient-CO_2_ environment. The findings suggest that carboxysome positioning by the McdAB system functions to maintain the carbon fixation efficiency of Rubisco by preventing carboxysome aggregation, which is particularly important under growth conditions where rod-shaped cyanobacteria adopt a filamentous morphology.

## INTRODUCTION

As the evolutionary ancestor of algae and plant chloroplasts, all cyanobacteria perform oxygenic photosynthesis and fix carbon dioxide through the Calvin-Benson-Bassham cycle. Unlike chloroplasts, cyanobacteria encapsulate their ribulose 1,5-bisphosphate carboxylase/oxidase (Rubisco) and carbonic anhydrase within large selectively permeable protein-based organelles called carboxysomes. This mechanism generates an environment around Rubisco that is significantly enriched in CO_2_, which increases the carboxylation activity of Rubisco, while simultaneously reducing photorespiration (1).

In the model rod-shaped cyanobacterium Synechococcus elongatus PCC 7942 (hereafter S. elongatus), carboxysomes were found to be uniformly distributed down the length of individual cells (2). This equidistant positioning supports equal inheritance of carboxysomes following cell division and may assist in maximizing the diffusion of substrates and products across the carboxysome shell. Carboxysomes are essential for the growth and survival of all cyanobacteria and are responsible for ∼35% of global carbon fixation through atmospheric CO_2_ assimilation (3, 4). However, it remains unknown how the subcellular organization of carboxysomes influences cyanobacterial physiology, a question of considerable ecological, evolutionary, and biotechnological importance.

Savage et al. were first to report that a ParA-type ATPase (hereafter McdA [for maintenance of carboxysome distribution A]) is required for positioning carboxysomes in *S. elongatus* (2). ParA family members have well-established roles in the segregation of bacterial chromosomes and plasmids (5, 6). Less studied are ParA family members shown to be required in the positioning of diverse protein complexes, such as those involved in secretion (7, 8), chemotaxis (9–11), conjugation (12), cell division (13, 14), and cell motility (15, 16), as well as bacterial microcompartments (BMCs), such as the carboxysome (2, 17). We recently identified a small novel protein, McdB, responsible for generating dynamic McdA gradients on the nucleoid (17). McdB colocalizes and directly interacts with carboxysomes and removes McdA from the nucleoid in their vicinity. We proposed that carboxysomes use a Brownian-ratchet mechanism whereby McdB-bound carboxysome motion occurs in a directed and persistent manner toward increased concentrations of McdA on the nucleoid. We also recently found that the McdAB system is widespread among β-cyanobacteria (18) and carbon-fixing proteobacteria (19). Together, the findings suggest that the equidistant positioning of carboxysomes in carbon-fixing bacteria is important, but the physiological consequences of carboxysome mispositioning in the absence of the McdAB system remain unclear.

When Savage et al. first identified the McdA requirement for carboxysome positioning, a minor decrease in CO_2_ fixation was found in a Δ*mcdA* strain, but growth at 30°C under ambient CO_2_ was the only condition studied (2). Rising atmospheric CO_2_ levels and sea surface temperatures are pervasive effects of climate change (20), and both have direct influences on Rubisco activity and cyanobacterial growth (21, 22). Here, we show that a failure to distribute carboxysomes leads to a temperature-dependent decrease in growth rate, cell elongation, asymmetric cell division, and elevated levels of cellular Rubisco. We also report that, unexpectedly, wild-type *S. elongatus* undergoes filamentous growth when switched from a high- to ambient-CO_2_ environment or when grown at 20°C, an environmentally relevant growth temperature for *S. elongatus* not commonly used in the lab. We propose that carboxysome positioning by the McdAB system functions as part of an autotrophic growth strategy that maintains the carbon fixation efficiency of Rubisco by preventing carboxysome aggregation. In the absence of carboxysome positioning, we propose the changes in cell growth, morphology, and Rubisco levels are all responses to organic carbon limitation.

## RESULTS

We performed *in vivo* microscopy to determine how carboxysome organization was altered in *mcdA*, *mcdB*, and *mcdAB* deletion strains compared to wild-type *S. elongatus*. Immunoblot analysis against McdA and McdB proteins verified the loss of expression from the deletion strains and verified that the remaining *mcdA* or *mcdB* gene was still expressed at levels similar to that of the wild type (see Fig. S1A in the supplemental material). In *S. elongatus*, each Rubisco enzyme is composed of eight large (RbcL) and eight small (RbcS) subunits to form RbcL_8_S_8_ (23). Therefore, to image carboxysomes, the fluorescent protein mTQ (monomeric Turquoise2) was fused to the C terminus of RbcS to make RbcS-mTQ (see Materials and Methods for strain construction details). *rbcS-mTQ* was expressed using a second copy of its native promoter (inserted at neutral site 1) in addition to wild-type *rbcS* at its native locus in wild-type cells as well as in the three deletion strains—Δ*mcdA*, Δ*mcdB*, and Δ*mcdAB* mutant strains. The presence of RbcS-mTQ did not alter growth rate (Fig. S1B to D). Carboxysomes of *S. elongatus* encapsulate between 800 to 1,500 copies of the Rubisco holoenzyme complex (24). Therefore, RbcS-mTQ provides a bright and high-contrast marker for quantifying the subcellular organization of carboxysomes, as previously shown (17). We also performed phase contrast imaging to monitor for potential changes in cell morphology and chlorophyll fluorescence imaging to verify that cells treated in our analyses were photosynthetically active and therefore viable (Fig. S2).

10.1128/mBio.02696-20.1 FIG S1 (A) Immunoblots against McdA and McdB proteins in wild-type and Δ*mcdA*, Δ*mcdB*, Δ*mcdAB* deletion strains. (B) Growth curves of wild-type *S. elongatus* with and without the RbcS-mTQ carboxysome marker. The RbcS-mTQ carboxysome marker does not influence growth. Cells were grown at 30°C in 2% CO_2_. (C) Comparison of growth rates calculated from growth curves in panel B. The unpaired *t* test shows growth rates were not statistically different. (D) As in panel B, but with the McdAB system deletion mutants. Error bars represent the standard deviations from three independent biological replicates. Download FIG S1, TIF file, 2.6 MB. Copyright © 2021 Rillema et al. 2021 Rillema et al. https://creativecommons.org/licenses/by/4.0/ This content is distributed under the terms of the Creative Commons Attribution 4.0 International license.

10.1128/mBio.02696-20.2 FIG S2 Phase contrast and chlorophyll autofluorescence imaging shows that the Δ*mcdA*, Δ*mcdB*, and Δ*mcdAB* deletion strains were photosynthetically active at all growth temperatures tested in this study. Cells were grown at the specified temperature in 2% CO_2_. Download FIG S2, TIF file, 2.6 MB. Copyright © 2021 Rillema et al. 2021 Rillema et al. https://creativecommons.org/licenses/by/4.0/ This content is distributed under the terms of the Creative Commons Attribution 4.0 International license.

In the lab, *S. elongatus* is typically grown at 32°C with a doubling time on the order of several hours depending on growth conditions (temperature, light, and CO_2_ availability). This was the temperature used in our previous study that identified the McdAB carboxysome positioning system (17). However, *S. elongatus* was recently shown to grow faster at 40°C (25, 26). Therefore, in anticipation of a growth defect in the absence of carboxysome positioning in our McdAB system mutants, we began our studies by growing cultures at 40°C in constant light under ambient (0.04%) or high (2%) CO_2_ concentrations.

### Carboxysome mispositioning correlates with minor defects in cellular physiology at 40°C.

**FIG 1:**
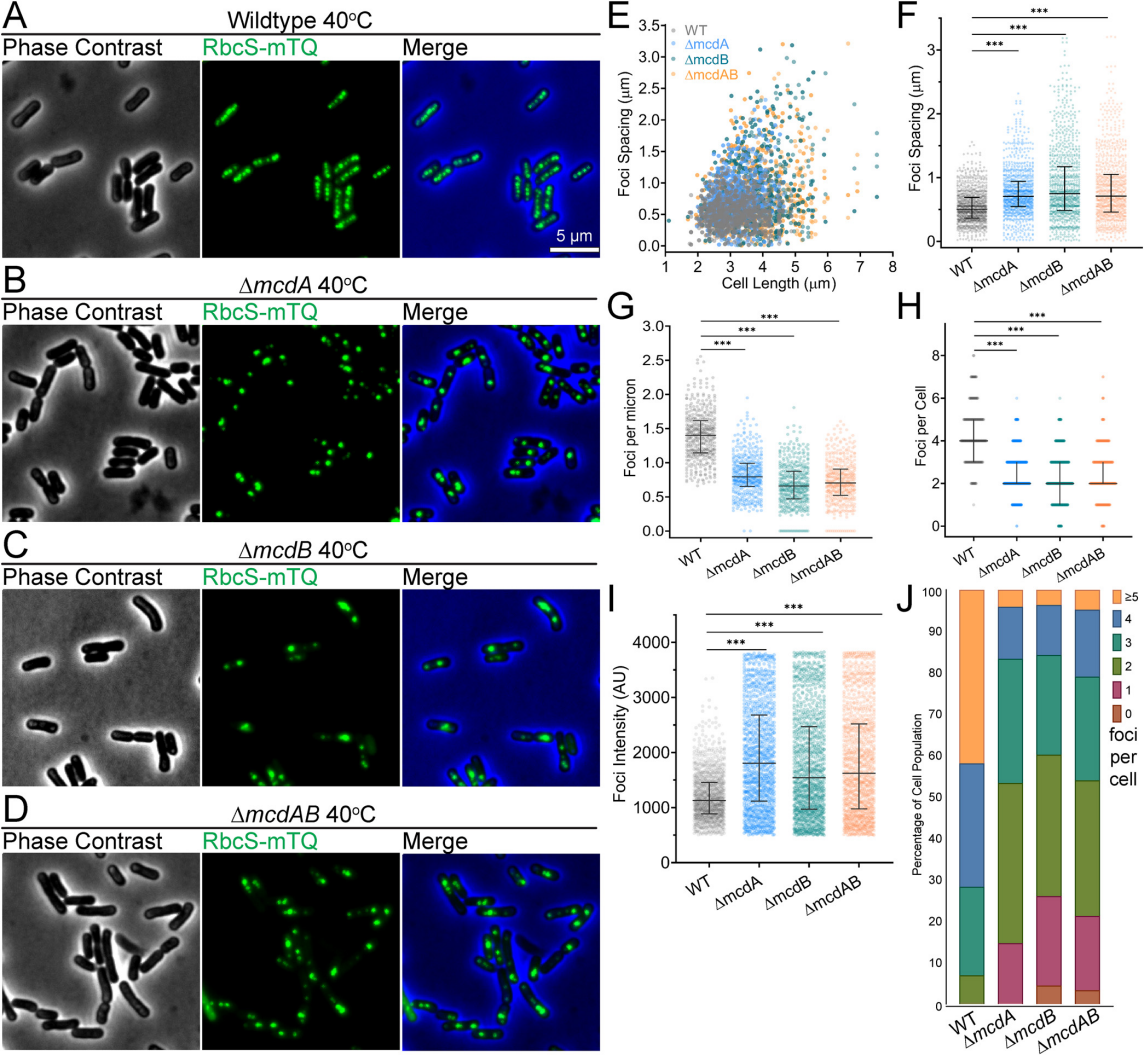
McdAB system mutants display fewer and mispositioned carboxysome aggregates at 40°C. (A to D) Microscopy images of the specified cell strains (wild type [WT] and Δ*mcdA*, Δ*mcdB*, and Δ*mcdAB* mutants) grown at 40°C in 2% CO_2_. Phase contrast micrographs are shown in black and white, and carboxysomes are shown in green. The phase contrast channel is blue in the merge. (E) Spacing between carboxysome foci as a function of cell length. (F) Spacing between carboxysome foci in the same cell. (G) Number of carboxysome foci per unit cell length. (H) Carboxysome foci number per cell. (I) Carboxysome peak focus intensity (in arbitrary units [AU]). (F to I) Solid bars represent the median and the 95% confidence interval. Statistical significance was based on a nonparametric Mann-Whitney test and is shown as follows: ***, *P* < 0.001. (J) Population percentages of cells with the specified number of carboxysome foci. *n* ≥ 1,000 carboxysomes from 440 cells of each strain.

We first verified that carboxysomes are organized by the McdAB system at 40°C, as we found previously at 32°C (17). We examined carboxysome positioning under high CO_2_ conditions (2% CO_2_) to maximize growth. We found that RbcS-mTQ-labeled carboxysomes were equally spaced down the long axis of wild-type *S. elongatus* cells (Fig. 1A). The Δ*mcdA*, Δ*mcdB*, and Δ*mcdAB* mutants lost this uniform positioning of carboxysomes (Fig. 1B to D), as observed previously at 32°C (17).

We compared the nearest neighbor spacing of carboxysome foci as a function of cell length (Fig. 1E). Wild-type cells showed uniform carboxysome spacing (0.55 ± 0.20 μm) regardless of cell length. All three mutants, on the other hand, displayed an increase in the nearest neighbor spacing of carboxysome foci as cell length increased. Both the median and variability in spacing were greater in the mutants compared to the wild type (Fig. 1F). The increased spacing resulted in fewer carboxysome foci per unit cell length (Fig. 1G), as well as fewer carboxysome foci per cell (Fig. 1H). When quantifying the carboxysome fluorescence intensity (Fig. 1I), it became apparent that the increased spacing in all three mutant populations was not likely a result of fewer carboxysomes being assembled, but rather, carboxysomes were coalescing into massive aggregates. Indeed, when comparing carboxysome focus number across cell populations, we find that ∼95% of wild-type cells (*n* = 440) had three or more foci, whereas ∼45% of all mutant populations had three or more foci (Fig. 1J). Roughly 20% of cells from all three mutant populations had a single carboxysome aggregate or no carboxysomes at all, whereas wild-type cells never had less than two foci. The 40°C data show that the McdAB system equally spaces carboxysomes down the cell length and also serves as an antiaggregator, preventing carboxysomes from coalescing.

**FIG 2:**
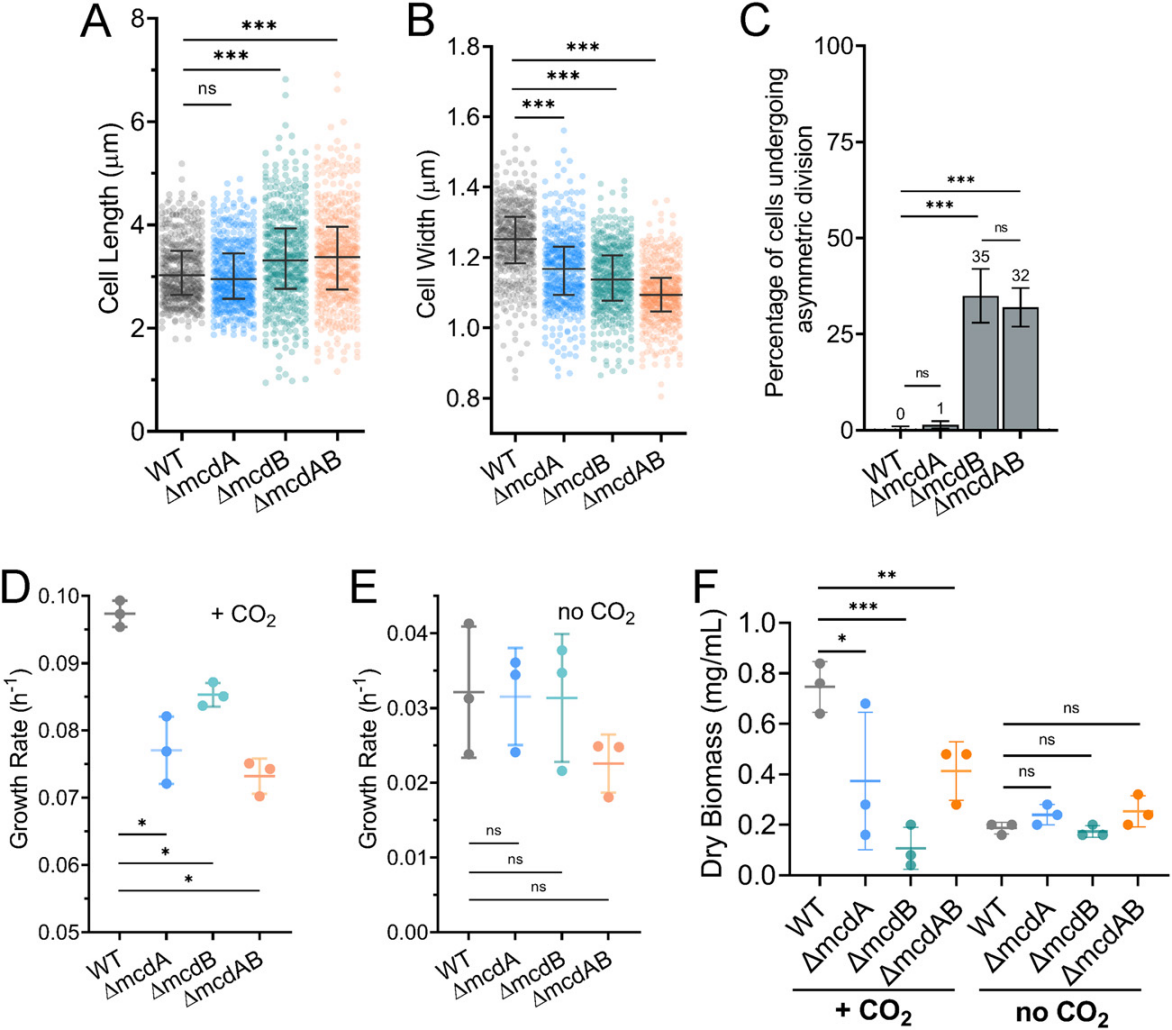
Carboxysome mispositioning correlates with subtle changes in cell physiology at 40°C. (A and B) Cell lengths (A) and widths (B) of the wild-type (WT) population compared to McdAB system mutants. Cells were grown at 40°C in 2% CO_2_. Statistical significance was based on a nonparametric Mann-Whitney test and shown as follows: ***, *P* < 0.001; n.s., not significant. (C) Percentage of cells undergoing asymmetric (non-mid-cell) division (*n* ≥ 440 dividing cells of each strain). (D and E) Quantification of mean exponential growth rate in 2% CO_2_ (D) or no CO_2_ added (E). (F) Quantification of dry cell biomass for cells grown in 2% CO_2_ or no CO_2_ added. In panels C to F, error bars represent the standard deviations from at least three independent biological replicates. Statistical significance was based on an unpaired *t* test and shown as follows: ***, *P* < 0.001; **, *P* < 0.01; *, *P* < 0.05; n.s., not significant.

We then asked whether carboxysome mispositioning affected cell physiology at 40°C. Wild-type *S. elongatus* cells were 3.1 ± 0.5 μm in length (Fig. 2A) and 1.25 ± 0.05 μm in width (Fig. 2B). We found that *ΔmcdA* cells were of a similar length but thinner compared to the wild-type cells (Fig. 2A and B). The *ΔmcdB* and *ΔmcdAB* cells had a slightly longer median cell length and wider distribution (Fig. 2A). These mutants were also thinner compared to the wild type (Fig. 2B). The presence of both longer and shorter cells suggested asymmetric cell division events in the *ΔmcdB* and *ΔmcdAB* cell populations. We quantified the frequency of symmetric (mid-cell) versus asymmetric (non-mid-cell) division events and found that asymmetric division was exclusive to the *ΔmcdB* and *ΔmcdAB* populations (Fig. 2C). The data show that while both McdA and McdB are required for positioning carboxysomes, the loss of McdB elicits an asymmetric division phenotype at 40°C.

The changes in cell morphology suggested that, even with high CO_2_ where carboxysomes are not essential for viability, carboxysome mispositioning may still alter cell growth. Interestingly, a reduction in growth rate was observed for all three mutants, but only when grown at high CO_2_ (Fig. 2D and F and Fig. S3A). When the cells were grown slowly in ambient CO_2_ (0.04%), we did not observe significant differences in growth rate compared to the wild type (Fig. 2E and F). Overall, at 40°C, carboxysomes are mispositioned in all three McdAB system mutants, with moderate increases in carboxysome spacing due to aggregation, which ultimately results in fewer carboxysome foci per cell. We also found an asymmetric cell division phenotype exclusive to strains lacking McdB. However, in all deletion strains, the growth rate was significantly lower than that of the wild type only when high CO_2_ was provided.

10.1128/mBio.02696-20.3 FIG S3 (A) Growth curves of all strains grown at 40°C in 2% CO_2_ or no CO_2_ added. (B) As in panel A, but cells grown at 30°C. (C) As in panel A, but cells grown at 20°C. Error bars represent the standard deviations from three independent biological replicates. Download FIG S3, TIF file, 2.4 MB. Copyright © 2021 Rillema et al. 2021 Rillema et al. https://creativecommons.org/licenses/by/4.0/ This content is distributed under the terms of the Creative Commons Attribution 4.0 International license.

### Carboxysome mispositioning causes cell elongation and asymmetric cell division at 30°C.

**FIG 3:**
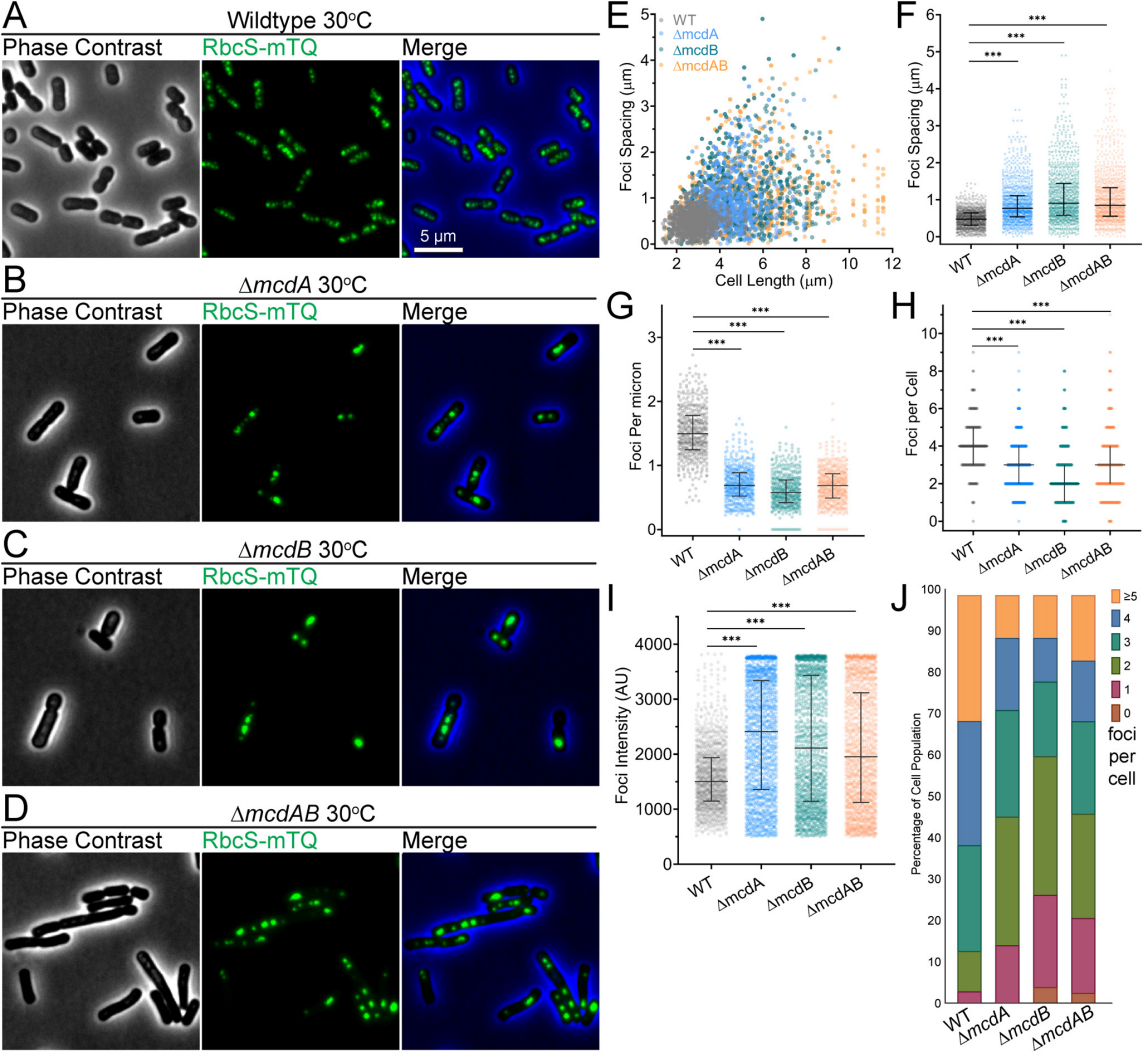
McdAB system mutants house few and mispositioned carboxysome aggregates at 30°C. (A to D) Microscopy images of the specified cell strains grown at 30°C in 2% CO_2_. Phase contrast micrographs are shown in black and white, and carboxysomes are shown in green. The phase contrast channel is blue in the merge. (E) Spacing between carboxysome foci as a function of cell length. (F) Spacing between carboxysome foci in the same cell. (G) Number of carboxysome foci per unit cell length. (H) Carboxysome foci number per cell. (I) Carboxysome peak foci intensity (in arbitrary units [AU]). (F to I) Solid bars represent the median and the 95% confidence interval. Statistical significance was based on a nonparametric Mann-Whitney test and indicated as follows: ***, *P* < 0.001. (J) Population percentages of cells with the specified number of carboxysome foci (*n* ≥ 1,000 carboxysomes from 440 cells of each strain).

We continued our study at 30°C, a growth temperature closer to what we used to first identify the McdAB carboxysome positioning system (17). As we showed previously, and similar to our 40°C data, wild-type *S. elongatus* cells have equally spaced carboxysomes (Fig. 3A), while in all three mutants, carboxysomes were mispositioned (Fig. 3B to D). Wild-type cells displayed the same carboxysome spacing distance (0.50 ± 0.20 μm) regardless of cell length, while all three mutants had increased carboxysome spacing and variability in spacing as cell length increased (Fig. 3E and F). Intriguingly, we found that mutant cell lengths were significantly longer compared to the wild-type length. Cell elongation was more extreme in the Δ*mcdB* and Δ*mcdAB* mutants, resulting in more distantly spaced carboxysome foci (Fig. 3F). The data once again show that McdB plays a currently unknown role in carboxysome function, separate from its role in carboxysome positioning with McdA.

The increased spacing resulted in fewer carboxysome foci per unit cell length (Fig. 3G) and fewer carboxysomes per cell (Fig. 3H). Carboxysome foci in all three mutants were significantly higher in intensity than that of wild type, consistent with aggregation (Fig. 3I). While ∼90% of wild-type cells (*n* = 486) had three or more foci, ∼50% of all three mutant populations had three or more foci (Fig. 3J). Again, ∼20% of cells in all three mutant populations contained a single carboxysome aggregate or no carboxysomes at all. This is a striking reduction in carboxysome foci when considering the cell elongation phenotype exclusive to the mutant cell lines.

**FIG 4:**
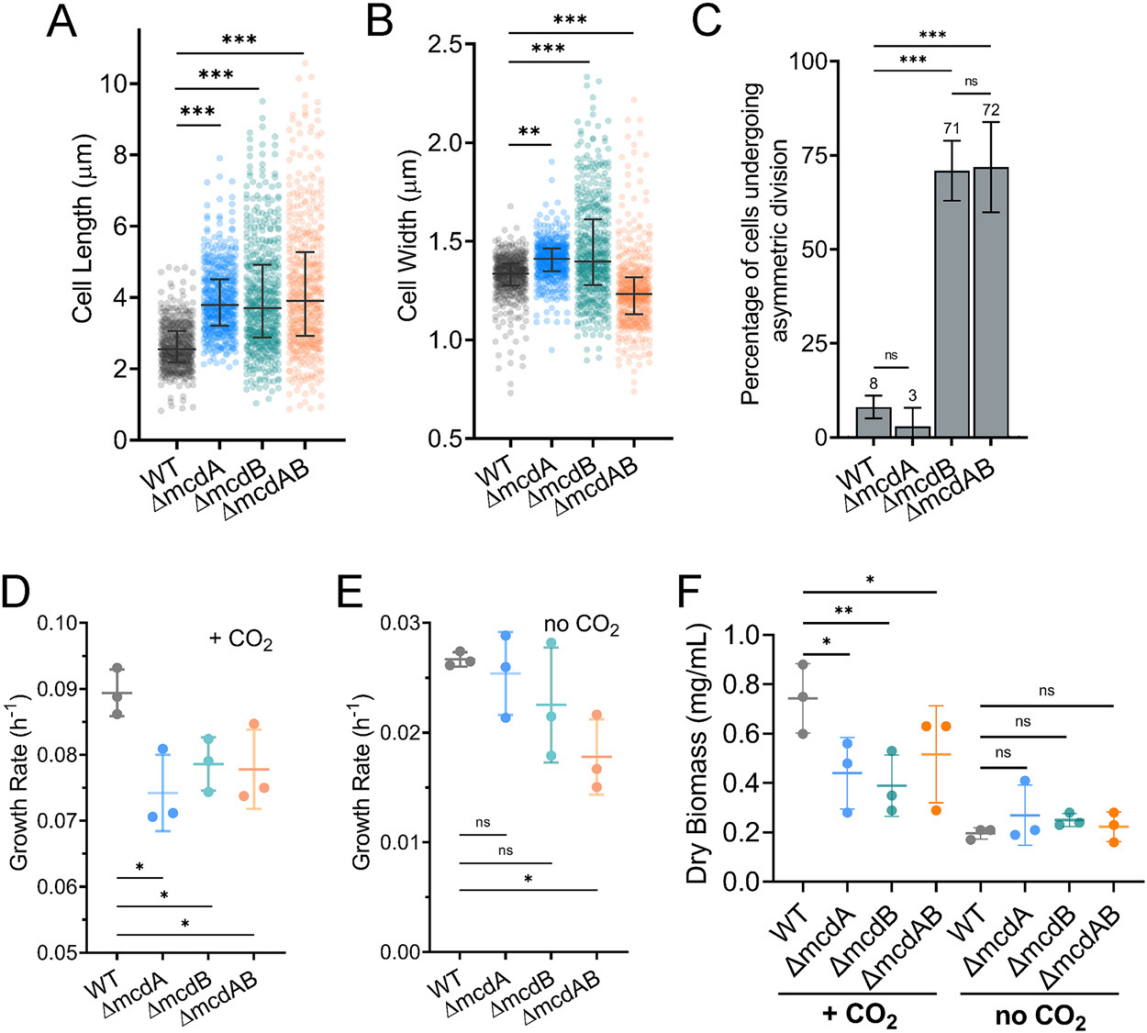
McdAB mutants display cell growth and morphology defects at 30°C. (A and B) Cell lengths (A) and widths (B) of WT population compared to McdAB system mutants. Cells were grown at 30°C in 2% CO_2_. Statistical significance was based on a nonparametric Mann-Whitney test and shown as follows: ***, *P* < 0.001; **, *P* < 0.01; n.s., not significant. (C) Percentage of cells undergoing asymmetric (non-mid-cell) division (*n* = 440 dividing cells of each strain). (D and E) Quantification of mean exponential growth rate in 2% CO_2_ (D) or no CO_2_ added (E). (F) Quantification of dry cell biomass for cells grown in 2% CO_2_ or no CO_2_ added. In panels C to F, error bars represent the standard deviations from at least three independent biological replicates. Statistical significance was based on an unpaired *t* test and indicated as follows: **, *P* < 0.01; *, *P* < 0.05; n.s., not significant.

As the carboxysome spacing data suggested, all three mutants had significantly longer cell lengths compared to the wild type when grown at 30°C (Fig. 4A). Median cell width was similar across all strains; however, *ΔmcdB* and *ΔmcdAB* cell populations displayed significantly wider distributions (Fig. 4B). Once again, the *ΔmcdB* and *ΔmcdAB* mutant populations displayed a significant number of asymmetrical division events (∼70% of dividing cells) compared to the wild-type or *ΔmcdA* strain (Fig. 4C). We propose that carboxysome mispositioning and aggregation at 30°C elicits cell division arrest, cell elongation, and when McdB is absent, asymmetric cell division; phenotypes that were less apparent when cells were grown at 40°C.

The cell elongation phenotype suggested that the mutants may also display lower growth rates, even when grown at high CO_2_. Indeed, with high CO_2_, a significant reduction in growth rate was observed for all three mutants compared to the wild type (Fig. 4D and F and Fig. S3B). However, once again, when cells were grown in ambient CO_2_, there was little to no reduction in growth rate compared to the wild type (Fig. 4E and F).

Overall, we find that at 30°C, carboxysomes are mispositioned in the *ΔmcdA*, *ΔmcdB*, and *ΔmcdAB* mutants with drastic increases in carboxysome spacing due to aggregation, resulting in fewer carboxysome foci per cell. We unveiled a cell elongation phenotype for all three mutant populations, and strikingly, we also found an asymmetric cell division phenotype that was exclusive to cells lacking McdB, a phenotype also found at 40°C but exacerbated at 30°C (compare Fig. 2C and 4C). Despite these carboxysome aggregation and cell morphology phenotypes, only minor decreases in growth rate were observed, and only under high CO_2_ conditions. We propose that carboxysome aggregation decreases the carbon-fixing activity of encapsulated Rubisco, which triggers a carbon limitation response that results in cell elongation and asymmetric cell division.

### Wild-type *S. elongatus* elongates at 20°C and growth is slower in McdAB system mutants.

A 10 degree drop in growth temperature unveiled the physiological consequences of carboxysome mispositioning at 30°C, which were largely absent at 40°C. The catalytic rate of carboxysome-encapsulated Rubisco is often considered the bottle neck of photosynthesis because the enzyme is inefficient and temperature dependent, whereas the light reactions of photosynthesis are temperature independent (27–29). *S. elongatus* PCC 7942 was originally isolated from a freshwater source in the San Francisco Bay area, with an annual temperature range of 8°C to 25°C (30). We therefore studied the effects of carboxysome mispositioning when cells were grown at 20°C, which is within the environmentally relevant temperature range.

**FIG 5:**
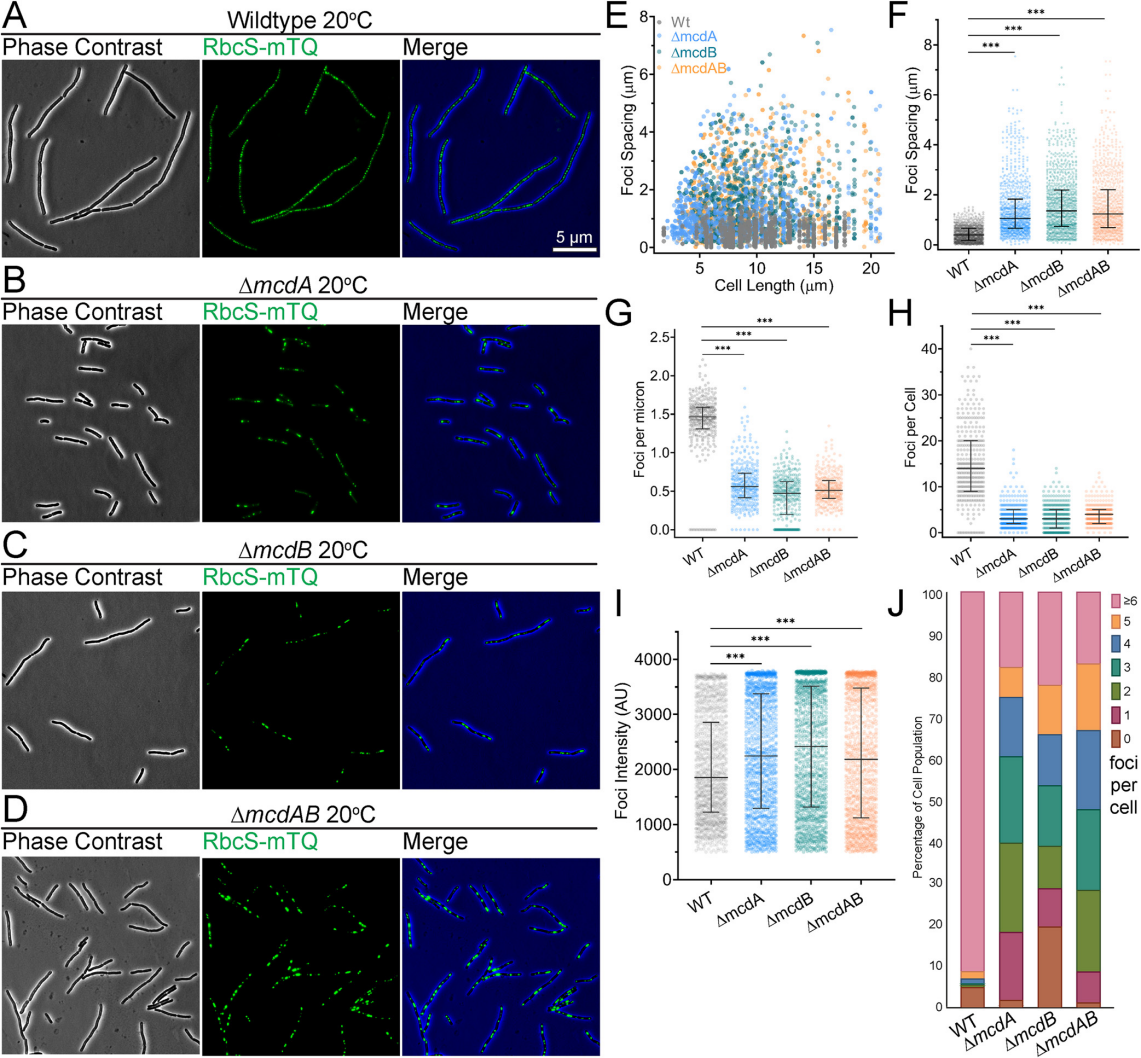
At 20°C, wild-type and McdAB system mutants elongate, but only the mutants display few and mispositioned carboxysome aggregates. (A to D) Microscopy images of the specified cell strains grown at 20°C in 2% CO_2_. Phase contrast micrographs are shown in black and white, and carboxysomes are shown in green. The phase contrast channel is blue in the merge. (E) Spacing between carboxysome foci as a function of cell length. (F) Spacing between carboxysome foci in the same cell. (G) Number of carboxysome foci per unit cell length. (H) Carboxysome foci number per cell. (I) Carboxysome peak foci intensity (in arbitrary units [AU]). (F to I) Solid bars represent the median and the 95% confidence interval. Statistical significance was based on a nonparametric Mann-Whitney test and indicated as follows: ***, *P* < 0.001. (J) Population percentages of cells with the specified number of carboxysome foci (*n* ≥ 1,000 carboxysomes from 440 cells of each strain).

Unexpectedly, we found that even wild-type *S. elongatus* undergoes extreme cell elongation when grown at 20°C (Fig. 5A). *S. elongatus* has been previously studied at this temperature (31), but to our knowledge, this is the first time its temperature-dependent cell elongation phenotype has been directly observed and reported. Despite the remarkably long cell lengths, carboxysomes were still well aligned down the entire longitudinal axis of the cell (Fig. 5A). All three mutants were also elongated, but to a lesser extent, and carboxysomes were clearly mispositioned (Fig. 5B to D).

The wild type showed the same uniform carboxysome spacing distance (0.50 ± 0.30 μm) even in cells as long as 20 μm (Fig. 5E). All three mutants, on the other hand, displayed dramatically increased carboxysome spacing and variability in spacing as cell length increased (Fig. 5F). Since all cell types dramatically elongated at 20°C, the loss of carboxysome positioning resulted in a massive reduction in the number of carboxysome foci per unit cell length (Fig. 5G) and per cell (Fig. 5H). Carboxysome foci in all three mutants were significantly larger than that of the wild type, once again suggesting aggregation (Fig. 5I). While ∼90% of wild-type cells (*n* = 388) had six or more foci, only ∼20% of all three mutant populations had six or more foci (Fig. 5J). The data emphasize the importance of the McdAB system in positioning carboxysomes in cells that are dramatically elongated when grown at cool, but environmentally relevant temperatures.

**FIG 6:**
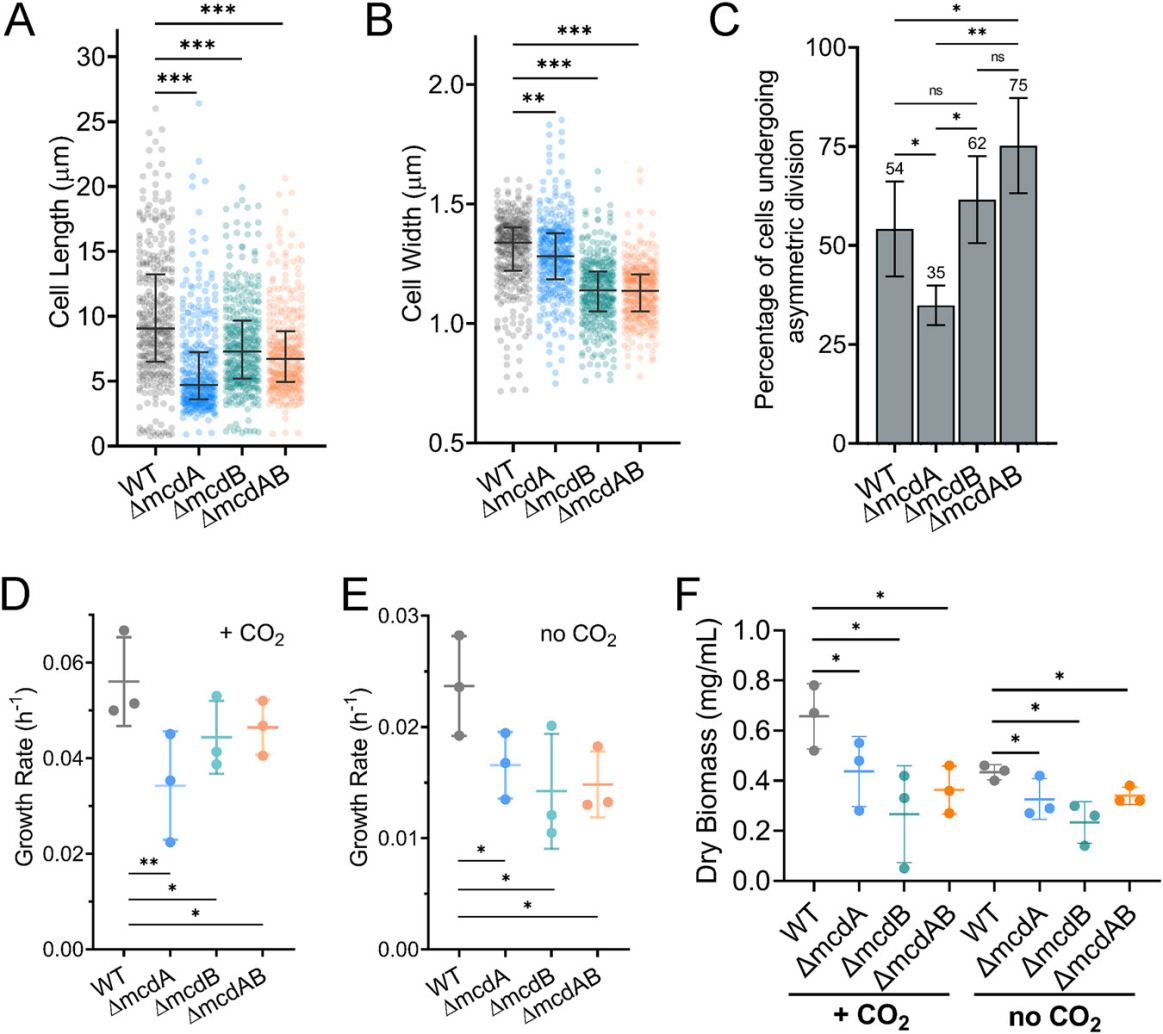
At 20°C, wildtype and McdAB mutants elongate and undergo asymmetric cell division, but only McdAB system mutants show lowered growth rates. (A and B) Cell lengths (A) and widths (B) of the WT population compared to McdAB system mutants. Cells were grown at 20°C in 2% CO_2_. Statistical significance was based on a nonparametric Mann-Whitney test and indicated as follows: ***, *P* < 0.001; **, *P* < 0.01. (C) Percentage of cells undergoing asymmetric (non-mid-cell) division (*n* ≥ 440 dividing cells of each strain). (D and E) Quantification of mean exponential growth rate in 2% CO_2_ (D) or no CO_2_ added (E). (F) Quantification of dry cell biomass for cells grown in 2% CO_2_ or no CO_2_ added. In panels C to F, error bars represent the standard deviations from at least three independent biological replicates. Statistical significance was based on an unpaired *t* test and indicated as follows: **, *P* < 0.01; *, *P* < 0.05; n.s., not significant.

We quantified cell length at 20°C and found that the median length of wild-type cells was significantly longer than those for all three mutant populations (Fig. 6A). The *ΔmcdA* cells were similar in width to that of the wild type, while *ΔmcdB* and *ΔmcdAB* cell populations were notably thinner (Fig. 6B). Consistent with the wide distributions in cell length (Fig. 6A), a significant fraction of division events at this growth temperature were asymmetric across all cell populations, including the wild type (Fig. 6C). However, the frequency of asymmetric division events was still highest in mutants lacking McdB. When growth rates were assayed at high CO_2_ (Fig. 6D and F) or ambient CO_2_ (Fig. 6E and F), we found statistically significant reductions in growth rate under both conditions for all three mutants compared to the wild type.

Overall, we identified a cell elongation phenotype in wild-type *S. elongatus* that occurs when grown at colder, but environmentally relevant temperatures. All three mutants elongated to a lesser extent and had aggregated carboxysomes, which resulted in drastically fewer carboxysome foci per cell compared to the wild type. Only at this cooler temperature, we found significant decreases in growth rate with or without CO_2_ added. We speculate that colder growth temperatures decrease the carbon fixation activity of Rubisco to a point where cell division arrest, elongation, and asymmetric cell division are triggered in response to carbon limitation, even in wild-type cells. At 30°C, this response was found only in the McdAB system mutants likely due to reduced carbon fixation efficiency resulting from carboxysome aggregation. At 40°C, higher Rubisco activity compensates for carboxysome aggregation, which would explain why McdAB system mutants displayed cell morphologies and growth rates closer to those of the wild type.

### McdAB mutants have elevated levels of cellular Rubisco.

**FIG 7:**
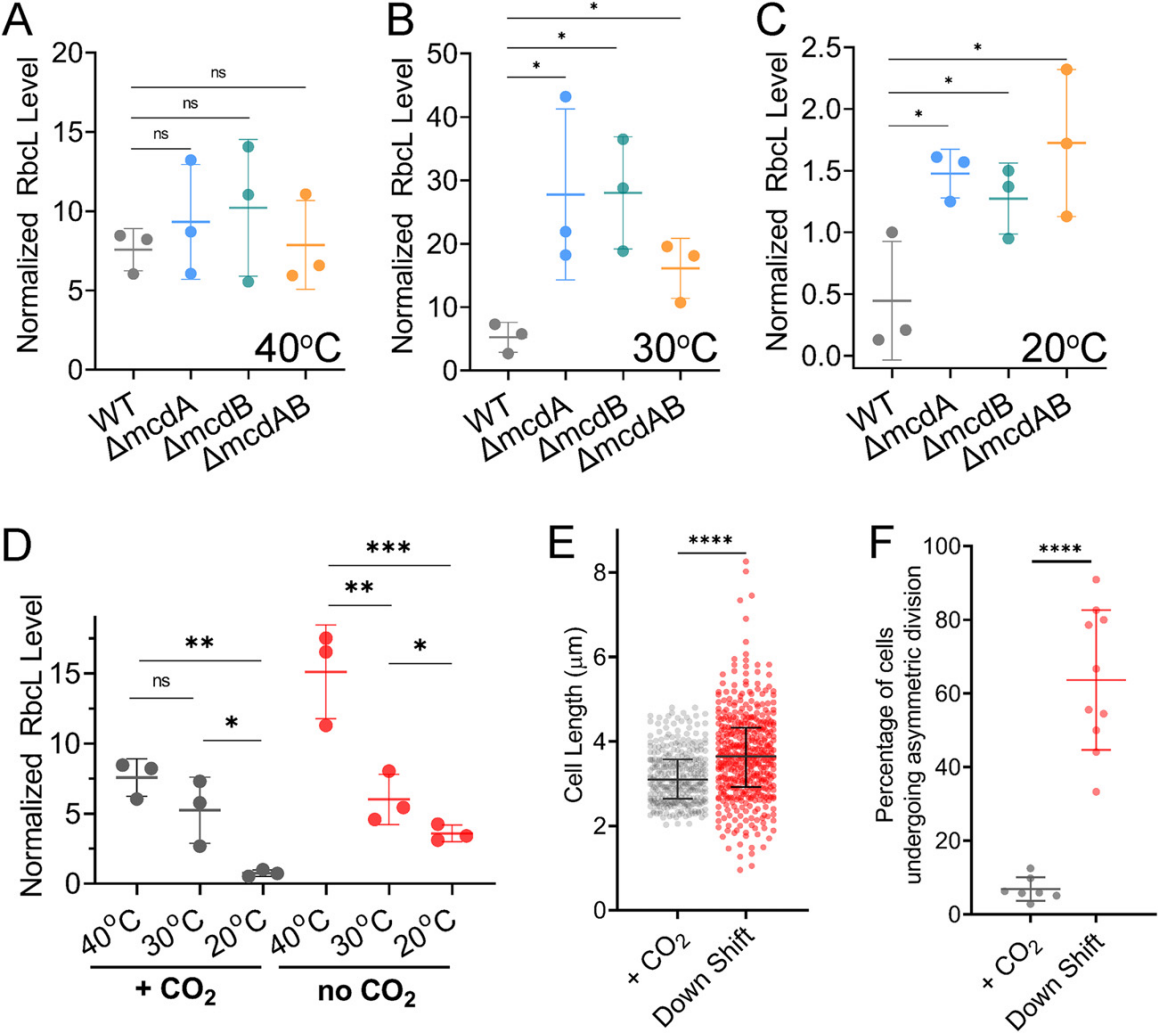
Increased levels of Rubisco correlate with cell elongation and asymmetric cell division phenotypes. (A) Quantification of immunoblots against the Rubisco large subunit (RbcL) in each strain grown at 40°C in 2% CO_2_. (B) As in panel A, but cells were grown at 30°C. (C) As in panel A, but cells were grown at 20°C. RbcL signals were normalized to Atpβ content. (D) Quantification of RbcL in wild-type *S. elongatus* cells grown at the indicated temperatures in 2% CO_2_ (gray) or no CO_2_ added (red). In panels A to D, error bars represent the standard deviations from three independent biological replicates. Statistical significance was based on an unpaired *t* test. (E) Cell length quantification of wild-type cells when maintained at 2% CO_2_ (+ CO_2_) or down-shifted to ambient CO_2_ (Down Shift). Wild-type *S. elongatus* cells elongate upon CO_2_ down-shift. Statistical significance was based on a nonparametric Mann-Whitney test (*n* ≥ 300 cells per condition). (F) Percentage of cells undergoing asymmetric division when maintained at 2% CO_2_ (+ CO_2_) or down-shifted to ambient CO_2_ (Down Shift). Wild-type *S. elongatus* undergoes asymmetric cell division upon CO_2_ down-shift. Error bars represent the standard deviations from at least three independent biological replicates (*n* ≥ 300 dividing cells). Each dot represents the value from an independent field of view (*n* ≥ 7 field of views per condition). Statistical significance was based on an unpaired *t* test and indicated as follows: ****, *P* < 0.0001; ***, *P* < 0.001; **, *P* < 0.01; *, *P* < 0.05; n.s., not significant.

The Rubisco encapsulated in carboxysomes is the sole enzyme providing organic carbon for cellular biomass production and the phototrophic growth of *S. elongatus*. We set out to determine whether carboxysome mispositioning and the changes in cell morphology of McdAB system mutants correlated with altered cellular levels of Rubisco using immunoblot analysis against the large subunit of Rubisco (RbcL) (Fig. S4). RbcL content from cell cultures was normalized based on Atpβ quantity from immunoblot analysis as shown previously (32, 33). We used high CO_2_ when assaying the McdAB system mutants, as this was the growth condition that showed the strongest physiological defects across all temperatures tested. At 40°C, Rubisco abundance in the mutant strains was not significantly different than that of wild type (Fig. 7A). However, at 30°C, Rubisco levels were three- to sixfold higher in all three mutants compared to the wild type (Fig. 7B). This trend was also observed at 20°C (Fig. 7C). Overall, our findings show that carboxysome aggregation correlates with a temperature-dependent increase in Rubisco abundance in the cell.

### Wild-type *S. elongatus* cells elongate, asymmetrically divide, and have elevated Rubisco levels in response to CO_2_ down-shift.

Finally, we set out to determine whether cell elongation and asymmetric cell division were consequences of reduced carbon fixation by performing CO_2_ down-shift assays on wild-type *S. elongatus*. Similar to our McdAB deletion mutants, we found that wild-type cells had increased levels of Rubisco at higher growth temperatures and when grown under ambient CO_2_ compared to when grown at high CO_2_ (Fig. 7D). The CO_2_ down-shift caused wild-type cells to elongate (Fig. 7E) and undergo asymmetric cell division, compared to cells maintained at high CO_2_ (Fig. 7F). Together, the data are consistent with our proposal that carboxysome aggregation in McdAB system mutants results in reduced carbon fixation activity by encapsulated Rubisco and a carbon limitation response—cell elongation, asymmetric cell division, and elevated levels of Rubisco.

## DISCUSSION

We recently identified the McdAB system that is responsible for the equidistant positioning of carboxysomes in the rod-shaped cyanobacterium *S. elongatus* PCC 7942 (17). We also recently found that McdAB systems are widespread among β-cyanobacteria (18) and carboxysome-containing proteobacteria (19). The findings suggest important and widespread, but currently unknown, physiological roles for carboxysome positioning in carbon-fixing bacteria. Here, we show that a failure to distribute carboxysomes in *S. elongatus* leads to several changes in cell physiology: slower growth, cell elongation, asymmetric cell division, and a significant increase in cellular levels of Rubisco.

At the three growth temperatures tested (20, 30, and 40°C), all three mutants housed few and irregularly spaced carboxysome aggregates, whereas wild-type cells have uniformly spaced and sized carboxysome foci. The data provide quantitative support for our previous finding that the McdAB system equally distributes carboxysomes to opposite sides of the cell to ensure inheritance following cell division (2, 17), akin to ParA-based plasmid partition systems in bacteria (5). However, in addition, and particularly important for protein-based cargoes, the McdAB system serves to prevent carboxysome aggregation. This “antiaggregation” activity serves as a homeostasis mechanism that regulates carboxysome size, number, composition, positioning, and ultimately, its carbon-fixing function in the cell. It has recently been found that the pyrenoid, the functional analog of the carboxysome in chloroplasts of the model alga *Chlamydomonas*, is also spatially regulated and this activity affects carbon fixation (34). It remains to be seen whether other BMCs also use spatial regulators to avoid aggregation during their self-assembly to optimize enzymatic efficiency.

### Physiological defects associated with carboxysome mispositioning are temperature dependent.

Despite the drastic mispositioning and aggregation of carboxysomes in the *mcdA*, *mcdB*, and *mcdAB* deletion strains of *S. elongatus*, only minor changes in cell morphology and growth rate were found at the highest temperature used in this study (40°C) (Fig. 2). At 30°C, however, all three mutants displayed a cell elongation phenotype, and a significant fraction of cell division events in the mutant populations lacking McdB were asymmetric (Fig. 4). Along with these changes in cell morphology, the growth rates of all three mutants were lower than that of wild type, but only at high CO_2_. At our coldest but environmentally relevant growth temperature of 20°C, we found that all cell populations, even the wild type, underwent filamentous growth and asymmetric cell division (Fig. 6). Wild-type cells at 20°C were as much as 10 times longer than the median length when grown at 30°C or 40°C. Even in these extremely elongated cells, the McdAB system robustly distributed carboxysomes down the entire cell length (Fig. 5A). This mode of filamentous growth was slower in all McdAB system mutants, resulting in significantly shorter filaments compared to the wild type (Fig. 6A). Overall, we found temperature-dependent physiological defects associated with carboxysome mispositioning and aggregation, phenotypes that were masked at higher temperatures typically used in the lab, and unveiled here at lower but environmentally relevant temperatures.

### Cell elongation and asymmetric cell division—a stress response to carbon limitation?

Many bacteria can change shape in response to growth conditions (35). Escherichia coli and Bacillus subtilis produce larger cells under nutrient-rich conditions and smaller cells under nutrient-limited conditions (36, 37), Pseudomonas aeruginosa elongates to enhance nutrient uptake during carbon and nitrogen starvation (38), Caulobacter crescentus cells increase cell area in response to phosphate starvation (39), and abundant human gut species such as Bacteroides thetaiotaomicron elongate under sugar-limited conditions (40). We propose that *S. elongatus* filamentation and asymmetric cell division are responses triggered by carbon limitation, resulting from reduced carbon fixation activity of Rubisco in this obligate photoautotroph. Cell filamentation (i) suppresses the birth of cells devoid of carboxysomes, (ii) gives the cell an opportunity to increase carbon fixation by producing more Rubisco, and (iii) increases cell surface area, which would increase the light-harvesting capability for photochemistry.

A carbon limitation response parsimoniously explains the three triggers identified here: (i) carboxysome aggregation, (ii) colder growth temperatures, and (iii) CO_2_ down-shift. For the carboxysome aggregation trigger, carbon limitation may result from a decrease in Rubisco efficiency due to losses in carboxysome integrity, substrate/product permeability, and/or enzyme stability. Our data suggest that all McdAB system mutants respond by significantly increasing the total cellular Rubisco content (Fig. 7B and C). We propose that this carbon limitation response can also be triggered in wild-type *S. elongatus* by low-temperature growth. At high temperature (i.e., 40°C), Rubisco activity may be sufficient to compensate for carboxysome aggregation in the McdAB system mutants. Therefore, the carbon limitation response is not triggered. At 30°C, Rubisco activity is still high enough to prevent a carbon limitation response in wild-type cells, but not in the McdAB system mutants with aggregated carboxysomes. As a result, cell filamentation is triggered only in the mutants. At 20°C, we speculate that Rubisco activity has reduced to a level that, even in wild-type cells, triggers a carbon limitation response. At this temperature, McdAB system mutants are starved for carbon to a point where growth is slowed, resulting in shorter filaments compared to the wild type (Fig. 6A). We were not able to perform *in vivo* carbon fixation assays to show a direct link between carbon fixation activity and the mutant phenotypes observed. However, we did find that wild-type *S. elongatus* cells elongate, asymmetrically divide, and increase Rubisco levels when shifting cultures from a high- to low-CO_2_ environment. The identical phenotypes implicate carbon limitation as the trigger for these physiological responses.

### Spatial regulation of carboxysomes influences Rubisco activity and abundance.

Previous studies have shown positive relationships between growth rate and Rubisco abundance (41–46). Elevated carbon fixation rates during blooms, for example, have been shown to be associated with severalfold increases in Rubisco. The 20°C temperature is well below the thermal optimum for Rubisco activity (47) and well below the optimal growth temperature of *S. elongatus*. We propose that the increased level of cellular Rubisco in the McdAB system mutants and the associated cell elongation phenotype both represent acclimation responses that compensate for the decreased activity of aggregated Rubisco at low-temperature growth. It is important to emphasize that cell elongation, lowered growth rate, and increased Rubisco levels were found when the cells were grown at high CO_2_—a condition whereby *S. elongatus* does not require the carbon-concentrating activity of carboxysomes for growth (48). This strengthens the argument that it is not CO_2_ substrate limitation causing these phenotypes. Instead, it is Rubisco activity that is significantly compromised when complexed in carboxysome aggregates. These findings highlight the physiological importance of carboxysome antiaggregation by the McdAB system in maximizing the carbon fixation activity of Rubisco.

Overall, we show that carboxysome distribution by the McdAB system in cyanobacteria can appropriately respond to variability in Rubisco abundance and activity along a wide temperature gradient. We speculate that this mechanism of carboxysome homeostasis provided by the McdAB system is part of an autotrophic growth strategy particularly in elongated cells—Rubisco is uniformly distributed into homogeneously sized carboxysomes at low temperature to overcome the lower activity of this temperature-dependent enzyme (46).

### McdB plays a role in carboxysome function outside of its positioning with McdA.

Regardless of growth temperature, Δ*mcdB* and Δ*mcdAB* mutants displayed a stronger asymmetric-division phenotype compared to the Δ*mcdA* mutant. The finding suggests that McdB plays a currently unknown but critical role in carboxysome function, outside of its role in positioning carboxysomes with McdA. Consistently, our recent bioinformatics analysis of McdAB systems across cyanobacteria (18) and proteobacteria (19) identified numerous species with orphan McdB proteins, once again suggesting a functional role independent of McdA. We have also shown previously via bacterial two-hybrid analysis that McdA does not physically associate with any carboxysome component, while McdB directly interacts with a number of shell proteins (17). We have also recently found that purified McdB undergoes liquid-liquid phase separation (LLPS) *in vitro* (18). This activity is intriguing given recent studies showing that both α- and β-carboxysomes, as well as the algal pyrenoid, have intrinsically disordered proteins that form liquid-like condensates with Rubisco (49–51). Collectively, these studies suggest that LLPS is a common feature underlying carboxysome biogenesis. The LLPS activity of McdB and other carboxysome components may be related, and potentially influence carboxysome function.

### Cold growth temperature triggers cell elongation and asymmetric cell division.

Across the bacterial world, complex systems maintain cell size homeostasis. We find here that wild-type *S. elongatus* maintains cell size homeostasis when grown at 30°C or 40°C but undergoes filamentation at the environmentally relevant temperature of 20°C. A growing number of bacterial species are known to elongate amid environmental changes to promote survival. For example, E. coli cells become filamentous during infection (52, 53), and during DNA damage, the SOS response blocks cell division until damage has been repaired (54). Several other forms of stress induce elongation, including host environment, antibiotics, nutrient access, pH, heat shock, and osmotic fluctuations (55–58). Bacteria can clearly exist in diverse morphological states, in part dictated by their environment (35, 59). To our knowledge, we find here the first example of bacterial filamentation caused by cold growth temperature. We propose colder temperatures trigger filamentation indirectly as a carbon limitation response due to the reduced carbon-fixing activity of Rubisco.

Alternatively, transition into a filamentous morphology has been proposed as a way to protect against predation in aquatic environments (56, 60–62). The growth temperature of 20°C coincides with the seasonal temperature during which predation occurs (61). Therefore, it is attractive to speculate that *S. elongatus* may use temperature as a cue to elongate so as to avoid planktivorous protists. Consistent with this possibility, previous studies have demonstrated that several freshwater bacteria, including C. crescentus (56), exhibit high phenotypic plasticity and can transition to a filamentous morphology—a transition that may be specifically triggered in the presence of a size-selective protistan predator (61, 63, 64). Therefore, some aquatic bacteria may undergo filamentation at temperatures that coincide with grazing season.

How bacterial cells enter and exit filamentous states to ensure survival during changes in environment remains poorly characterized. We find here that *S. elongatus* elongates when grown at low temperature or when carboxysomes are mispositioned. When these filaments divide, it is asymmetric, with one daughter cell of “normal” length. *S. elongatus* also elongates under dim-light stress, and then divides asymmetrically to form daughter cells of the correct size when brought back into well-lit conditions (65). The Min system has been shown to play a role in the asymmetric cell division and daughter cell sizing of filamentous E. coli (58), Vibrio parahaemolyticus (66, 67), and *S. elongatus* (65). Division restoration at the poles of these filaments has recently been shown to be regulated by a combination of Min oscillations, FtsZ levels and *terminus* segregation, resulting in daughter cells of the right length (68, 69). It should be noted that in addition to sensing light and inorganic carbon substrates, *S. elongatus* responds to lowering temperatures by slowing and even turning off circadian rhythms, thereby downregulating cell growth, division, and metabolic flux (70, 71). The mechanism by which filamentation and asymmetric division occurs in *S. elongatus* is an area of future research. Taken together, the conserved ability for various bacterial species to undergo filamentation and morphological recovery, some of which showed direct selective benefits, strongly suggests that this differentiation plays an important role in survival and proliferation.

## MATERIALS AND METHODS

### Construct designs.

All constructs in this study were generated using Gibson Assembly (72) from synthetized double-stranded DNA (dsDNA) and verified by sequencing. Constructs contained flanking DNA that ranged from 500 to 1,500 bp in length upstream and downstream of the targeted insertion site to promote homologous recombination into target genomic loci (73).

### Generation of bacterial strains.

All Synechococcus elongatus PCC 7942 transformations were performed as previously described (73). Strains used in this study are shown in Table 1. Plasmid constructs of *mcdA*, *mcdB*, and *mcdAB* deletions were created by replacing the respective coding sequences with a kanamycin resistance cassette. All fluorescent strains were transformed using plasmid pAH40, which contains a chloramphenicol resistance cassette and a second copy of the *rbcS* promoter and gene, attached at the 3′ end with the gene encoding fluorescent protein mTurquoise2 (mTQ) and separated with a GSGSGS linker, inserted into neutral site 1. Transformed cells were plated on BG-11 agar. Single colonies were picked into 96-well plates containing 300 μl of BG-11 with 6.35 μg ml^−1^ kanamycin or 3.125 μg ml^−1^ chloramphenicol. Concentrations of both respective antibiotics were gradually increased to 12.5 μg ml^−1^. Cultures were verified for complete insertion via PCR and removed from antibiotics for experiments.

**TABLE 1:** Synechococcus elongatus PCC 7942 strains used in this study

Strain	Description	Reference
Wild type		
Δ*mcdA*		17
Δ*mcdB*		17
Δ*mcdAB*		17
*rbcS-mTQ*	Wild type transformed with pAH40	
Δ*mcdA* + *rbcS-mTQ*	Δ*mcdA* mutant transformed with pAH40	
Δ*mcdB* + *rbcS-mTQ*	Δ*mcdB* mutant transformed with pAH40	
Δ*mcdAB* + *rbcS-mTQ*	Δ*mcdAB* mutant transformed with pAH40	

### Growth conditions.

Both wild-type and mutant Synechococcus elongatus PCC 7942 strains were grown in 125-ml culture flasks (Corning) in 50 ml BG-11 (Sigma) medium buffered with 1 g liter^−1^ HEPES to pH 8.3 shaken at 130 rpm or on BG-11 plates containing 1.5% (wt/vol) agar. All strains were maintained and grown under constant light-emitting dioide (LED) illumination of 60 μE m^−2^ s^−1^ at either 20°C, 30°C, or 40°C in 2% or 0.04% CO_2_ as specified. Cultures grown in 0.04% CO_2_ (ambient) were grown in air with no additional CO_2_. Cultures were regularly diluted with fresh medium to maintain exponential growth phase for subsequent imaging and immunoblot analyses. For cloning, One Shot TOP10 Chemically Competent E. coli (ThermoFisher) were grown aerobically at 37° in Luria broth medium.

### Fluorescence microscopy.

Microscopy was performed using exponentially growing cells at an optical density (OD) of 0.4. Two milliliters of culture was spun down at 15,000 × *g* for 60 s and resuspended in 100 μl of BG-11. Five microliters was transferred to a square 1.5% agarose plus BG-11 pad, which was then flipped onto a 35-mm cell culture dish with a #1.5 glass coverslip bottom (ManTek). All images were captured using a Nikon Eclipse Ti2 inverted microscope with a PlanApo Objective lens (100×, 1.45 numerical aperture [NA], oil immersion), with phase contrast trans-illumination, and with a SOLA LED light source for imaging chlorophyll fluorescence (excitation, 560/40 nm [540 to 580 nm]; emission, 630/70 nm [593 to 668 nm], dichroic mirror, 585 nm) and RbcS-mTQ fluorescence (excitation, 436/20 nm [426 to 446 nm]; emission, 480/40 nm [460 to 500 nm]; dichroic mirror, 455 nm). Images were acquired using a Photometrics Prime 95B back-illuminated scientific complementary metal oxide semiconductor (sCMOS) camera. Image analysis was performed using Fiji (74) and the MicrobeJ plugin (75).

### Growth curve measurements.

Cultures were inoculated at a starting optical density at 750 nm (OD_750_) of 0.1 to 0.2 with fresh BG-11. Cell growth was monitored at OD_750_ using a DS-11 spectrophotometer (Denovi) at the specified time points. Growth rates were calculated using the linear portion of the exponential phase of growth (<0.8 OD) to prevent shading effects on OD measurements. Error bars represent the standard deviations from three biological replicates that were recorded from different culture flasks.

### Biomass measurements.

All strains were inoculated at a starting OD_750_ of 0.1 for 24 h (at 30 and 40°C), 48 h (at 20°C with 2% CO_2_), or 10 days (at 20°C with ambient CO_2_). Cultures were filtered using MF-Millipore membrane filter, 0.22-μm pore size (catalog no. JGWP04700; Sigma-Aldrich). Membrane filters with cells were dried at 80°C for 20 h. Biomass was calculated by subtracting the weight of a membrane filter without cells (60 mg) from the weight of dried membrane filters with cells.

### Microbe quantification.

Multiple fields of view were taken for each cell strain using three channels: phase contrast microscopy provided cell length, width, and perimeter, while fluorescence microscopy provided chlorophyll autofluorescence and RbcS-mTQ intensities. Chlorophyll autofluorescence was used to determine the site of cell division in dividing cells. These data were analyzed using MicrobeJ (75). In each cell line, cell length detection was performed using the rod-shaped descriptor and thresholding set to 0.4 μm < area < maximum, 0.71 μm < width range < 2 μm, 0 μm < width variation < 0.2 μm, and 0 μm < angularity amplitude < 0.35 μm. Carboxysome detection was performed using the point function with a tolerance of 20 and an intensity minimum of 500. Associations, shape descriptors, profiles, and distances were recorded for each strain. All MicrobeJ quantification was also verified manually. Graphs and statistical analyses were generated with Graph Pad Prism.

### Immunoblot analysis.

Cultures were concentrated to an OD_750_ of 3 when harvesting. An equal volume of 2× Laemmli sample buffer was added prior to boiling for 20 min. Samples (55 μl) were loaded on a 4% to 12% Bis-Tris NuPAGE gel with wedge wells (Invitrogen). Gels were transferred onto a mini-size polyvinylidene difluoride membrane (Bio-Rad) using a Trans-Blot Turbo system (Bio-Rad). The membrane was immunoprobed using rabbit polyclonal antisera against RbcL and the beta subunit of ATP synthase, Atpβ (Agrisera) and then horseradish peroxidase (HRP)-conjugated anti-rabbit IgG secondary antibody (Millipore Sigma). Membrane signals were developed with femto maximum sensitivity substrate (Thermo Scientific) and visualized and quantified using Li-Cor Image Studio (three biological replicates). We normalized RbcL signal using Atpβ, a method previously used in Zhang et al. (33) and specifically with *S. elongatus* PCC 7942 in Sun et al. (32).

### CO_2_ down-shift assay.

Wild-type *S. elongatus* cultures were grown at 30°C with 2% CO_2_ until reaching OD_750_ of 0.6. Cultures were spun down, and the spent medium was discarded. Cell pellets were resuspended in fresh BG-11 medium and split into equal volumes to continue growth at 30°C with 2% CO_2_ or ambient CO_2_. After 72 h, phase contrast images of ≥300 cells for each condition from ≥5 fields of view were collected to quantify cell length and asymmetric cell division.

10.1128/mBio.02696-20.4 FIG S4 Representative immunoblots against RbcL protein in wild-type and Δ*mcdA*, Δ*mcdB*, and Δ*mcdAB* deletion strains at the growth temperature indicated. Quantification of immunoblots from at least three independent replicates were quantified and presented in Fig. 7A to C. Download FIG S4, TIF file, 2.7 MB. Copyright © 2021 Rillema et al. 2021 Rillema et al. https://creativecommons.org/licenses/by/4.0/ This content is distributed under the terms of the Creative Commons Attribution 4.0 International license.

## References

[1] Rae BD, Long BM, Badger MR, Price GD. 2013. Functions, compositions, and evolution of the two types of carboxysomes: polyhedral microcompartments that facilitate CO_2_ fixation in cyanobacteria and some proteobacteria. Microbiol Mol Biol Rev 77:357–379. doi:10.1128/MMBR.00061-12. 24006469 PMC3811607

[2] Savage DF, Afonso B, Chen AH, Silver PA. 2010. Spatially ordered dynamics of the bacterial carbon fixation machinery. Science 327:1258–1261. doi:10.1126/science.1186090. 20203050

[3] Cohen Y, Gurevitz M. 2006. The cyanobacteria—ecology, physiology and molecular genetics, p 1074–1098. In Dworkin M, Falkow S, Rosenberg E, Schleifer K-H, Stackebrandt E (ed), The prokaryotes, vol 4. Bacteria: Firmicutes, Cyanobacteria. Springer US, New York, NY.

[4] Kerfeld CA, Melnicki MR. 2016. Assembly, function and evolution of cyanobacterial carboxysomes. Curr Opin Plant Biol 31:66–75. doi:10.1016/j.pbi.2016.03.009. 27060669

[5] Baxter JC, Funnell BE. 2014. Plasmid partition mechanisms. Microbiol Spectr . doi:10.1128/microbiolspec.PLAS-0023-2014. 26104442

[6] Badrinarayanan A, Le TBK, Laub MT. 2015. Bacterial chromosome organization and segregation. Annu Rev Cell Dev Biol 31:171–199. doi:10.1146/annurev-cellbio-100814-125211. 26566111 PMC4706359

[7] Viollier PH, Sternheim N, Shapiro L. 2002. Identification of a localization factor for the polar positioning of bacterial structural and regulatory proteins. Proc Natl Acad Sci U S A 99:13831–13836. doi:10.1073/pnas.182411999. 12370432 PMC129783

[8] Perez-Cheeks BA, Planet PJ, Sarkar IN, Clock SA, Xu Q, Figurski DH. 2012. The product of tadZ, a new member of the parA/minD superfamily, localizes to a pole in Aggregatibacter actinomycetemcomitans. Mol Microbiol 83:694–711. doi:10.1111/j.1365-2958.2011.07955.x. 22239271 PMC3305808

[9] Thompson SR, Wadhams GH, Armitage JP. 2006. The positioning of cytoplasmic protein clusters in bacteria. Proc Natl Acad Sci U S A 103:8209–8214. doi:10.1073/pnas.0600919103. 16702547 PMC1472454

[10] Ringgaard S, Schirner K, Davis BM, Waldor MK. 2011. A family of ParA-like ATPases promotes cell pole maturation by facilitating polar localization of chemotaxis proteins. Genes Dev 25:1544–1555. doi:10.1101/gad.2061811. 21764856 PMC3143943

[11] Alvarado A, Kjær A, Yang W, Mann P, Briegel A, Waldor MK, Ringgaard S. 2017. Coupling chemosensory array formation and localization. Elife 6:e31058. doi:10.7554/eLife.31058. 29058677 PMC5706961

[12] Atmakuri K, Cascales E, Burton OT, Banta LM, Christie PJ. 2007. Agrobacterium ParA/MinD-like VirC1 spatially coordinates early conjugative DNA transfer reactions. EMBO J 26:2540–2551. doi:10.1038/sj.emboj.7601696. 17505518 PMC1868908

[13] Raskin DM, De Boer PAJ. 1999. Rapid pole-to-pole oscillation of a protein required for directing division to the middle of Escherichia coli. Proc Natl Acad Sci U S A 96:4971–4976. doi:10.1073/pnas.96.9.4971. 10220403 PMC21801

[14] Jordan A, Chandler J, MacCready JS, Huang J, Osteryoung KW, Ducat DC. 2017. Engineering cyanobacterial cell morphology for enhanced recovery and processing of biomass. Appl Environ Microbiol 83:e00053-17. doi:10.1128/AEM.00053-17. 28235875 PMC5394314

[15] Youderian P, Burke N, White DJ, Hartzell PL. 2003. Identification of genes required for adventurous gliding motility in Myxococcus xanthus with the transposable element mariner. Mol Microbiol 49:555–570. doi:10.1046/j.1365-2958.2003.03582.x. 12828649

[16] Kusumoto A, Shinohara A, Terashima H, Kojima S, Yakushi T, Homma M. 2008. Collaboration of FlhF and FlhG to regulate polar-flagella number and localization in Vibrio alginolyticus. Microbiology (Reading) 154:1390–1399. doi:10.1099/mic.0.2007/012641-0. 18451048

[17] MacCready JS, Hakim P, Young EJ, Hu L, Liu J, Osteryoung KW, Vecchiarelli AG, Ducat DC. 2018. Protein gradients on the nucleoid position the carbon-fixing organelles of cyanobacteria. Elife 7:e39723. doi:10.7554/eLife.39723. 30520729 PMC6328274

[18] MacCready JS, Basalla JL, Vecchiarelli AG. 2020. Origin and evolution of carboxysome positioning systems in cyanobacteria. Mol Biol Evol 37:1434–1451. doi:10.1093/molbev/msz308. 31899489 PMC7182216

[19] MacCready JS, Tran L, Basalla JL, Hakim P, Vecchiarelli AG. 2021. The McdAB system positions α-carboxysomes in proteobacteria. Mol Microbiol doi:10.1111/mmi.14708. PMC8359340 33638215

[20] Doney SC, Ruckelshaus M, Emmett Duffy J, Barry JP, Chan F, English CA, Galindo HM, Grebmeier JM, Hollowed AB, Knowlton N, Polovina J, Rabalais NN, Sydeman WJ, Talley LD. 2012. Climate change impacts on marine ecosystems. Annu Rev Mar Sci 4:11–37. doi:10.1146/annurev-marine-041911-111611. 22457967

[21] Eppley R. 1972. Temperature and phytoplankton growth in the sea. Fish Bull Natl Ocean Atmos Admin 70:1063–1085.

[22] Chen B, Liu H, Huang B, Wang J. 2014. Temperature effects on the growth rate of marine picoplankton. Mar Ecol Prog Ser 505:37–47. doi:10.3354/meps10773.

[23] Tabita FR, Hanson TE, Satagopan S, Witte BH, Kreel NE. 2008. Phylogenetic and evolutionary relationships of RubisCO and the RubisCO-like proteins and the functional lessons provided by diverse molecular forms. Philos Trans R Soc B 363:2629–2640. doi:10.1098/rstb.2008.0023. PMC2606765 18487131

[24] Sun Y, Wollman AJM, Huang F, Leake MC, Liu L-N. 2019. Single-organelle quantification reveals stoichiometric and structural variability of carboxysomes dependent on the environment. Plant Cell 31:1648–1664. doi:10.1105/tpc.18.00787. 31048338 PMC6635877

[25] Ohbayashi R, Nakamachi A, Hatakeyama TS, Watanabe S, Kanesaki Y, Chibazakura T, Yoshikawa H, Miyagishima S. 2019. Coordination of polyploid chromosome replication with cell size and growth in a cyanobacterium. mBio 10:e00510-19. doi:10.1128/mBio.00510-19. 31015323 PMC6478999

[26] Yu J, Liberton M, Cliften PF, Head RD, Jacobs JM, Smith RD, Koppenaal DW, Brand JJ, Pakrasi HB. 2015. Synechococcus elongatus UTEX 2973, a fast growing cyanobacterial chassis for biosynthesis using light and CO_2_. Sci Rep 5:8132. doi:10.1038/srep08132. 25633131 PMC5389031

[27] Erb TJ, Zarzycki J. 2018. A short history of RubisCO: the rise and fall (?) of Nature’s predominant CO2 fixing enzyme. Curr Opin Biotechnol 49:100–107. doi:10.1016/j.copbio.2017.07.017. 28843191 PMC7610757

[28] Geider RJ. 1987. Light and temperature dependence of the carbon to chlorophyll a ratio in microalgae and cyanobacteria: implications for physiology and growth of phytoplankton. New Phytol 106:1–34. doi:10.1111/j.1469-8137.1987.tb04788.x.

[29] Ensminger I, Busch F, Huner NPA. 2006. Photostasis and cold acclimation: sensing low temperature through photosynthesis. Physiol Plant 126:28–44. doi:10.1111/j.1399-3054.2006.00627.x.

[30] Welkie DG, Rubin BE, Diamond S, Hood RD, Savage DF, Golden SS. 2019. A hard day’s night: cyanobacteria in diel cycles. Trends Microbiol 27:231–242. doi:10.1016/j.tim.2018.11.002. 30527541 PMC6377297

[31] Burns RA, MacDonald CD, McGinn PJ, Campbell DA. 2006. Inorganic carbon repletion disrupts photosynthetic acclimation to low temperature in the cyanobacterium Synechococcus elongatus. J Phycol 42:610–621. doi:10.1111/j.1529-8817.2005.04101.x.

[32] Sun Y, Casella S, Fang Y, Huang F, Faulkner M, Barrett S, Liu LN. 2016. Light modulates the biosynthesis and organization of cyanobacterial carbon fixation machinery through photosynthetic electron flow. Plant Physiol 171:530–541. doi:10.1104/pp.16.00107. 26956667 PMC4854705

[33] Zhang P, Eisenhut M, Brandt AM, Carmel D, Silén HM, Vass I, Allahverdiyeva Y, Salminen TA, Aro EM. 2012. Operon flv4-flv2 provides cyanobacterial photosystem II with flexibility of electron transfer. Plant Cell 24:1952–1971. doi:10.1105/tpc.111.094417. 22570444 PMC3442580

[34] Itakura AK, Chan KX, Atkinson N, Pallesen L, Wang L, Reeves G, Patena W, Caspari O, Roth R, Goodenough U, McCormick AJ, Griffiths H, Jonikas MC. 2019. A Rubisco-binding protein is required for normal pyrenoid number and starch sheath morphology in Chlamydomonas reinhardtii. Proc Natl Acad Sci U S A 116:18445–18454. doi:10.1073/pnas.1904587116. 31455733 PMC6744930

[35] Kysela DT, Randich AM, Caccamo PD, Brun YV. 2016. Diversity takes shape: understanding the mechanistic and adaptive basis of bacterial morphology. PLoS Biol 14:e1002565. doi:10.1371/journal.pbio.1002565. 27695035 PMC5047622

[36] Sargent MG. 1975. Control of cell length in Bacillus subtilis. J Bacteriol 123:7–19. doi:10.1128/jb.123.1.7-19.1975. 806582 PMC235685

[37] Jun S, Si F, Pugatch R, Scott M. 2018. Fundamental principles in bacterial physiology—history, recent progress, and the future with focus on cell size control: a review. Rep Prog Phys 81:e056601. doi:10.1088/1361-6633/aaa628. PMC5897229 29313526

[38] Steinberger RE, Allen AR, Hansa HG, Holden PA. 2002. Elongation correlates with nutrient deprivation in Pseudomonas aeruginosa unsaturated biofilms. Microb Ecol 43:416–423. doi:10.1007/s00248-001-1063-z. 12043001

[39] Gonin M, Quardokus EM, O’Donnol D, Maddock J, Brun YV. 2000. Regulation of stalk elongation by phosphate in Caulobacter crescentus. J Bacteriol 182:337–347. doi:10.1128/JB.182.2.337-347.2000. 10629178 PMC94281

[40] Rangarajan AA, Koropatkin NM, Biteen JS. 2020. Nutrient-dependent morphological variability of Bacteroides thetaiotaomicron. Microbiology (Reading) 166:624–628. doi:10.1099/mic.0.000924. 32416743

[41] Falkowski PG, Sukenik A, Herzig R. 1989. Nitrogen limitation in Isochrysis galbana (Haptophyceae). II. Relative abundance of chloroplast proteins. J Phycol 25:471–478. doi:10.1111/j.1529-8817.1989.tb00252.x.

[42] Raven JA, Johnston AM. 1991. Mechanisms of inorganic‐carbon acquisition in marine phytoplankton and their implications for the use of other resources. Limnol Oceanogr 36:1701–1714. doi:10.4319/lo.1991.36.8.1701.

[43] Losh JL, Morel FMM, Hopkinson BM. 2012. Modest increase in the C:N ratio of N-limited phytoplankton in the California Current in response to high CO_2_. Mar Ecol Prog Ser 468:31–42. doi:10.3354/meps09981.

[44] Losh JL, Young JN, Morel FMM. 2013. Rubisco is a small fraction of total protein in marine phytoplankton. New Phytol 198:52–58. doi:10.1111/nph.12143. 23343368

[45] Fernández-González C, Pérez-Lorenzo M, Pratt N, Moore CM, Bibby TS, Marañón E. 2020. Effects of temperature and nutrient supply on resource allocation, photosynthetic strategy, and metabolic rates of Synechococcus sp. J Phycol 56:818–829. doi:10.1111/jpy.12983. 32130730

[46] Young JN, Goldman JAL, Kranz SA, Tortell PD, Morel FMM. 2015. Slow carboxylation of Rubisco constrains the rate of carbon fixation during Antarctic phytoplankton blooms. New Phytol 205:172–181. doi:10.1111/nph.13021. 25283055

[47] Galmés J, Aranjuelo I, Medrano H, Flexas J. 2013. Variation in Rubisco content and activity under variable climatic factors. Photosynth Res 117:73–90. doi:10.1007/s11120-013-9861-y. 23748840

[48] Cameron JC, Wilson SC, Bernstein SL, Kerfeld CA. 2013. Biogenesis of a bacterial organelle: the carboxysome assembly pathway. Cell 155:1131–1140. doi:10.1016/j.cell.2013.10.044. 24267892

[49] Oltrogge LM, Chaijarasphong T, Chen AW, Bolin ER, Marqusee S, Savage DF. 2020. Multivalent interactions between CsoS2 and Rubisco mediate α-carboxysome formation. Nat Struct Mol Biol 27:281–287. doi:10.1038/s41594-020-0387-7. 32123388 PMC7337323

[50] Wang H, Yan X, Aigner H, Bracher A, Nguyen ND, Hee WY, Long BM, Price GD, Hartl FU, Hayer-Hartl M. 2019. Rubisco condensate formation by CcmM in beta-carboxysome biogenesis. Nature 566:131–135. doi:10.1038/s41586-019-0880-5. 30675061

[51] Freeman Rosenzweig ES, Xu B, Kuhn Cuellar L, Martinez-Sanchez A, Schaffer M, Strauss M, Cartwright HN, Ronceray P, Plitzko JM, Förster F, Wingreen NS, Engel BD, Mackinder LCM, Jonikas MC. 2017. The eukaryotic CO_2_-concentrating organelle is liquid-like and exhibits dynamic reorganization. Cell 171:148–162.e19. doi:10.1016/j.cell.2017.08.008. 28938114 PMC5671343

[52] Horvath DJ, Li B, Casper T, Partida-Sanchez S, Hunstad DA, Hultgren SJ, Justice SS. 2011. Morphological plasticity promotes resistance to phagocyte killing of uropathogenic Escherichia coli. Microbes Infect 13:426–437. doi:10.1016/j.micinf.2010.12.004. 21182979 PMC3071881

[53] Justice SS, Hung C, Theriot JA, Fletcher DA, Anderson GG, Footer MJ, Hultgren SJ. 2004. Differentiation and developmental pathways of uropathogenic Escherichia coli in urinary tract pathogenesis. Proc Natl Acad Sci U S A 101:1333–1338. doi:10.1073/pnas.0308125100. 14739341 PMC337053

[54] Kreuzer KN. 2013. DNA damage responses in prokaryotes: regulating gene expression, modulating growth patterns, and manipulating replication forks. Cold Spring Harb Perspect Biol 5:a012674. doi:10.1101/cshperspect.a012674. 24097899 PMC3809575

[55] Justice SS, Hunstad DA, Cegelski L, Hultgren SJ. 2008. Morphological plasticity as a bacterial survival strategy. Nat Rev Microbiol 6:162–168. doi:10.1038/nrmicro1820. 18157153

[56] Heinrich K, Leslie DJ, Morlock M, Bertilsson S, Jonas K. 2019. Molecular basis and ecological relevance of Caulobacter cell filamentation in freshwater habitats. mBio 10:e01557-19. doi:10.1128/mBio.01557-19. 31431551 PMC6703425

[57] Bos J, Zhang Q, Vyawahare S, Rogers E, Rosenberg SM, Austin RH. 2015. Emergence of antibiotic resistance from multinucleated bacterial filaments. Proc Natl Acad Sci U S A 112:178–183. doi:10.1073/pnas.1420702111. 25492931 PMC4291622

[58] Wehrens M, Ershov D, Rozendaal R, Walker N, Schultz D, Kishony R, Levin PA, Tans SJ. 2018. Size laws and division ring dynamics in filamentous Escherichia coli cells. Curr Biol 28:972–979.e5. doi:10.1016/j.cub.2018.02.006. 29502951

[59] Yang DC, Blair KM, Salama NR. 2016. Staying in shape: the impact of cell shape on bacterial survival in diverse environments. Microbiol Mol Biol Rev 80:187–203. doi:10.1128/MMBR.00031-15. 26864431 PMC4771367

[60] Wortinger MA, Quardokus EM, Brun YV. 1998. Morphological adaptation and inhibition of cell division during stationary phase in Caulobacter crescentus. Mol Microbiol 29:963–973. doi:10.1046/j.1365-2958.1998.00959.x. 9767565

[61] Pernthaler J, Zöllner E, Warnecke F, Jürgens K. 2004. Bloom of filamentous bacteria in a mesotrophic lake: identity and potential controlling mechanism. Appl Environ Microbiol 70:6272–6281. doi:10.1128/AEM.70.10.6272-6281.2004. 15466575 PMC522086

[62] Pernthaler J. 2005. Predation on prokaryotes in the water column and its ecological implications. Nat Rev Microbiol 3:537–546. doi:10.1038/nrmicro1180. 15953930

[63] Hahn MW, Moore ERB, Höfle MG. 1999. Bacterial filament formation, a defense mechanism against flagellate grazing, is growth rate controlled in bacteria of different phyla. Appl Environ Microbiol 65:25–35. doi:10.1128/AEM.65.1.25-35.1999. 9872755 PMC90978

[64] Simek K, Vrba J, Pernthaler J, Posch T, Hartman P, Nedoma J, Psenner R. 1997. Morphological and compositional shifts in an experimental bacterial community influenced by protists with contrasting feeding modes. Appl Environ Microbiol 63:587–595. doi:10.1128/aem.63.2.587-595.1997. 16535515 PMC1389521

[65] Liao Y, Rust MJ. 2018. The Min oscillator defines sites of asymmetric cell division in cyanobacteria during stress recovery. Cell Syst 7:471–481.e6. doi:10.1016/j.cels.2018.10.006. 30414921

[66] Muraleedharan S, Freitas C, Mann P, Glatter T, Ringgaard S. 2018. A cell length-dependent transition in MinD-dynamics promotes a switch in division-site placement and preservation of proliferating elongated Vibrio parahaemolyticus swarmer cells. Mol Microbiol 109:365–384. doi:10.1111/mmi.13996. 29885061

[67] MacCready JS, Vecchiarelli AG. 2018. In long bacterial cells, the Min system can act off-center. Mol Microbiol 109:268–272. doi:10.1111/mmi.13995. 29885047

[68] Raghunathan S, Chimthanawala A, Krishna S, Vecchiarelli AG, Badrinarayanan A. 2020. Asymmetric chromosome segregation and cell division in DNA damage-induced bacterial filaments. Mol Biol Cell 31:2920–2931. doi:10.1091/mbc.E20-08-0547. 33112716 PMC7927188

[69] Cayron J, Dedieu A, Lesterlin C. 6 March 2020. Bacterial filament division dynamics allows rapid post-stress cell proliferation. bioRxiv 10.1101/2020.03.16.993345. 36527185

[70] Broddrick JT, Welkie DG, Jallet D, Golden SS, Peers G, Palsson BO. 2019. Predicting the metabolic capabilities of Synechococcus elongatus PCC 7942 adapted to different light regimes. Metab Eng 52:42–56. doi:10.1016/j.ymben.2018.11.001. 30439494 PMC6407710

[71] Murayama Y, Kori H, Oshima C, Kondo T, Iwasaki H, Ito H. 2017. Low temperature nullifies the circadian clock in cyanobacteria through Hopf bifurcation. Proc Natl Acad Sci U S A 114:5641–5646. doi:10.1073/pnas.1620378114. 28515313 PMC5465896

[72] Gibson DG, Young L, Chuang RY, Venter JC, Hutchison CA, Smith HO. 2009. Enzymatic assembly of DNA molecules up to several hundred kilobases. Nat Methods 6:343–345. doi:10.1038/nmeth.1318. 19363495

[73] Clerico EM, Ditty JL, Golden SS. 2007. Specialized techniques for site-directed mutagenesis in cyanobacteria. Methods Mol Biol 362:155–171. doi:10.1007/978-1-59745-257-1_11. 17417008

[74] Schindelin J, Arganda-Carreras I, Frise E, Kaynig V, Longair M, Pietzsch T, Preibisch S, Rueden C, Saalfeld S, Schmid B, Tinevez J-Y, White DJ, Hartenstein V, Eliceiri K, Tomancak P, Cardona A. 2012. Fiji: an open-source platform for biological-image analysis. Nat Methods 9:676–682. doi:10.1038/nmeth.2019. 22743772 PMC3855844

[75] Ducret A, Quardokus EM, Brun YV. 2016. MicrobeJ, a tool for high throughput bacterial cell detection and quantitative analysis. Nat Microbiol 1:16077. doi:10.1038/nmicrobiol.2016.77. 27572972 PMC5010025

