# Supplementary figures and images for: Carboxysome Mispositioning Alters Growth, Morphology, and Rubisco Level of the Cyanobacterium Synechococcus elongatus PCC 7942
Source: mBio. 2021 Aug 3;12(4):e02696-20. doi: 10.1128/mBio.02696-20 (PMC8406218; doi:10.1128/mBio.02696-20)

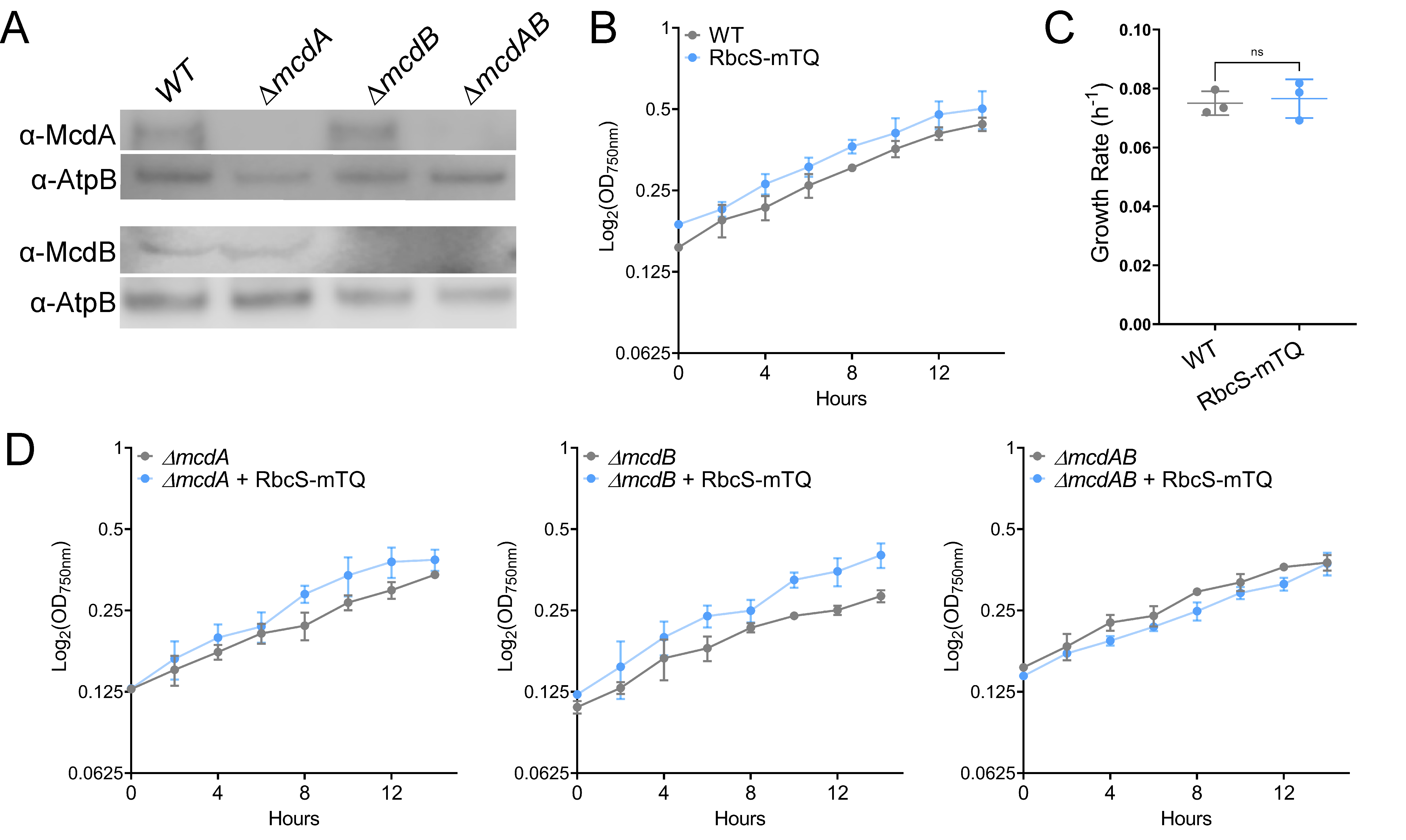

Supplement: FIG S1 [file mbio.02696-20-sf001.tif]

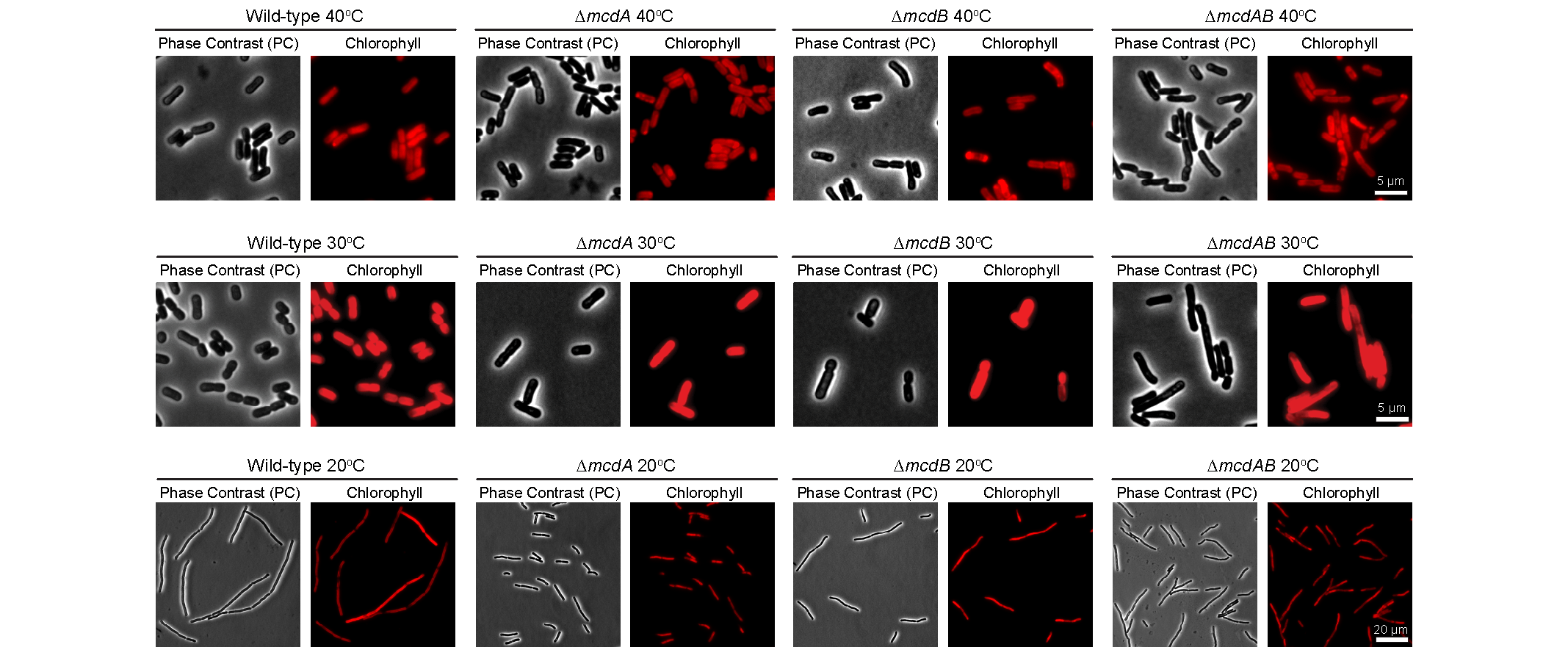

Supplement: FIG S2 [file mbio.02696-20-sf002.tif]

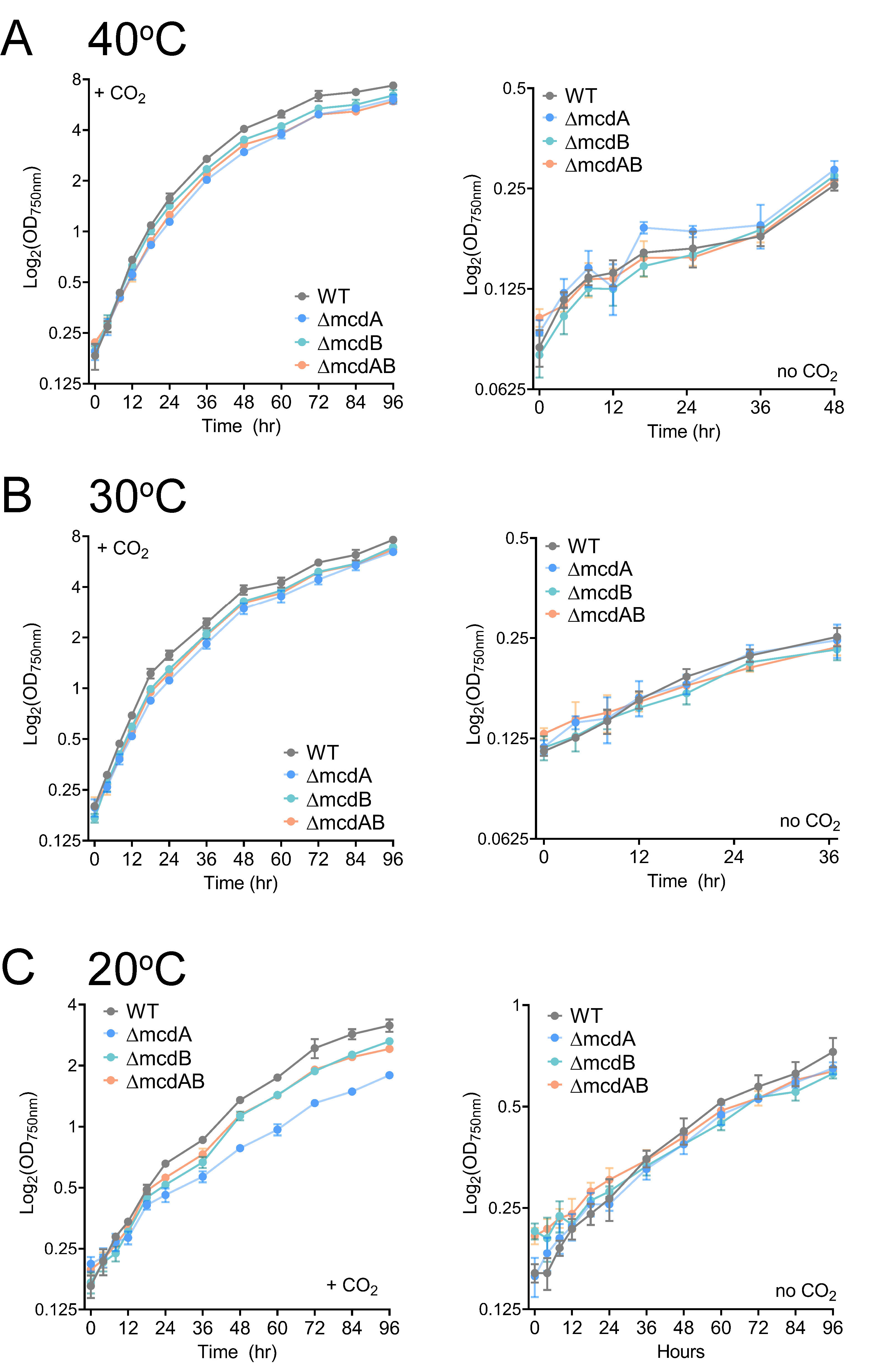

Supplement: FIG S3 [file mbio.02696-20-sf003.tif]

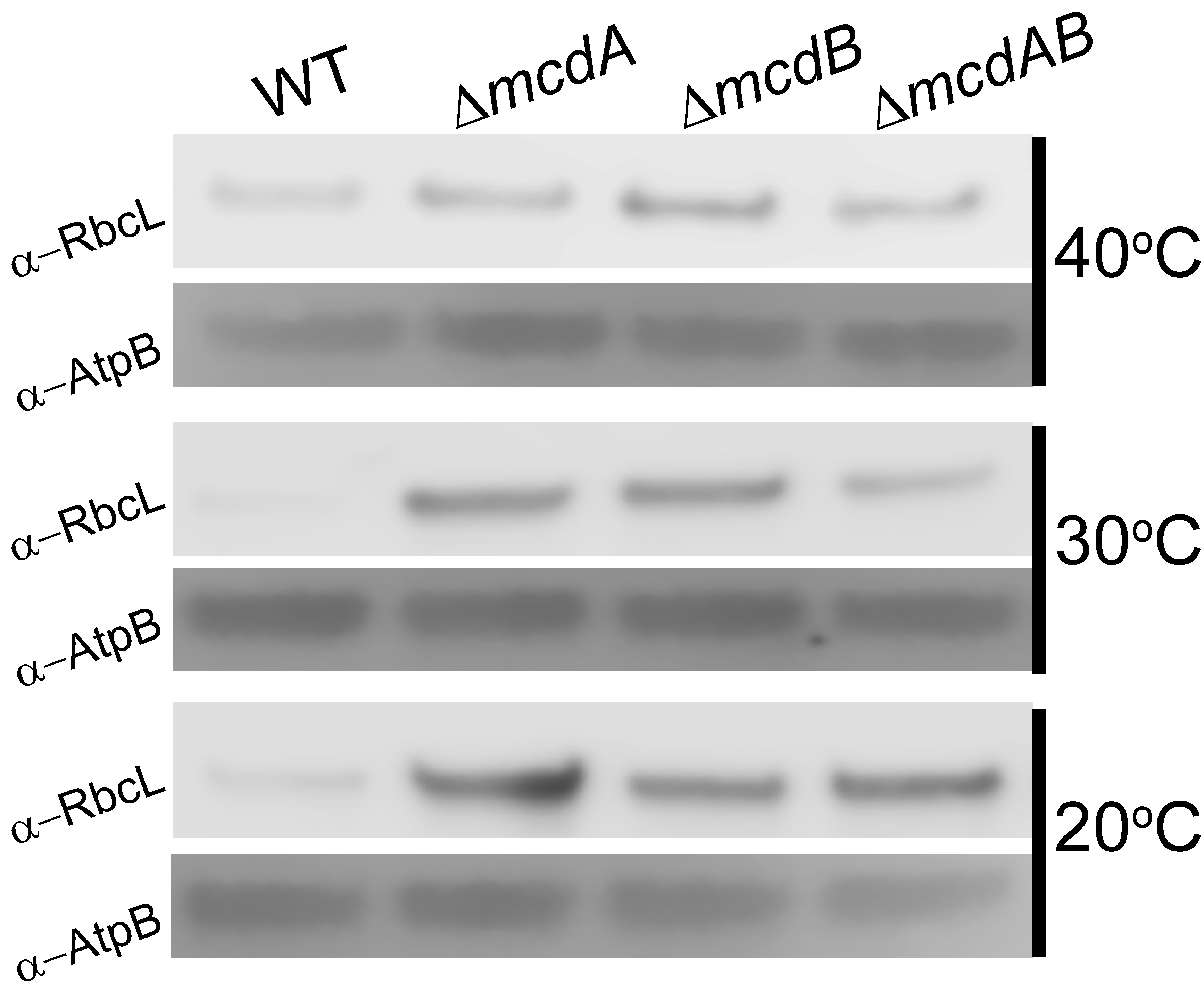

Supplement: FIG S4 [file mbio.02696-20-sf004.tif]
